# Cell therapy using ex vivo reprogrammed macrophages enhances antitumor immune responses in melanoma

**DOI:** 10.1186/s13046-024-03182-w

**Published:** 2024-09-14

**Authors:** Satish kumar Reddy Noonepalle, Maria Gracia-Hernandez, Nima Aghdam, Michael Berrigan, Hawa Coulibaly, Xintang Li, Christian Zevallos-Delgado, Andrew Pletcher, Bryan Weselman, Erica Palmer, Tessa Knox, Eduardo Sotomayor, Katherine B. Chiappinelli, Duncan Wardrop, Anelia Horvath, Brett A. Shook, Norman Lee, Anatoly Dritschilo, Rohan Fernandes, Karthik Musunuri, Maho Shibata, Alejandro Villagra

**Affiliations:** 1 The George Washington University, Washington, DC USA; 2 Tampa General Hospital, Tampa, FL USA; 3 University of Illinois at Chicago, Chicago, IL USA; 4 Avstera Therapeutics, Malvern, PA USA; 5 Lombardi Comprehensive Cancer Center, Georgetown University, 3970 Reservoir Road, NW, E416 Research Bldg, Washington, DC 20057 USA

## Abstract

**Background:**

Macrophage-based cell therapies have shown modest success in clinical trials, which can be attributed to their phenotypic plasticity, where transplanted macrophages get reprogrammed towards a pro-tumor phenotype. In most tumor types, including melanoma, the balance between antitumor M1-like and tumor-promoting M2-like macrophages is critical in defining the local immune response with a higher M1/M2 ratio favoring antitumor immunity. Therefore, designing novel strategies to increase the M1/M2 ratio in the TME has high clinical significance and benefits macrophage-based cell therapies.

**Methods:**

In this study, we reprogrammed antitumor and proinflammatory macrophages ex-vivo with HDAC6 inhibitors (HDAC6i). We administered the reprogrammed macrophages intratumorally as an adoptive cell therapy (ACT) in the syngeneic SM1 murine melanoma model and patient-derived xenograft bearing NSG-SGM3 humanized mouse models. We phenotyped the tumor-infiltrated immune cells by flow cytometry and histological analysis of tumor sections for macrophage markers. We performed bulk RNA-seq profiling of murine bone marrow-derived macrophages treated with vehicle or HDAC6i and single-cell RNA-seq profiling of SM1 tumor-infiltrated immune cells to determine the effect of intratumor macrophage ACT on the tumor microenvironment (TME). We further analyzed the single-cell data to identify key cell-cell interactions and trajectory analysis to determine the fate of tumor-associated macrophages post-ACT.

**Results:**

Macrophage ACT resulted in diminished tumor growth in both mouse models. We also demonstrated that HDAC6 inhibition in macrophages suppressed the polarization toward tumor-promoting phenotype by attenuating STAT3-mediated M2 reprogramming. Two weeks post-transplantation, ACT macrophages were viable, and inhibition of HDAC6 rendered intratumor transplanted M1 macrophages resistant to repolarization towards protumor M2 phenotype in-vivo. Further characterization of tumors by flow cytometry, single-cell transcriptomics, and single-cell secretome analyses revealed a significant enrichment of antitumor M1-like macrophages, resulting in increased M1/M2 ratio and infiltration of CD8 effector T-cells. Computational analysis of single-cell RNA-seq data for cell-cell interactions and trajectory analyses indicated activation of monocytes and T-cells in the TME.

**Conclusions:**

In summary, for the first time, we demonstrated the potential of reprogramming macrophages ex-vivo with HDAC6 inhibitors as a viable macrophage cell therapy to treat solid tumors.

**Supplementary Information:**

The online version contains supplementary material available at 10.1186/s13046-024-03182-w.

## Introduction

The tumor microenvironment (TME) is a complex ecosystem of interplay between cancer cells and immune cells [1]. Tumor-associated macrophages (TAMs), the most abundant immune cells in the TME, play an essential role in shaping innate and adaptive antitumor immunity [2]. Macrophages can be classified into proinflammatory M1-like and anti-inflammatory M2-like phenotypes. However, TAMs can switch between these phenotypes or exhibit a spectrum of hybrid characteristics [3]. The dichotomous terminology of M1 and M2 macrophages does not truly represent the macrophage phenotype and function of TAMs within the TME. Meta-analysis of single-cell transcriptomics data revealed multiple TAM subsets based on gene expression signatures classified into interferon primed TAMs, immune regulatory TAMs, inflammatory cytokines enriched TAMs, lipid-associated TAMs, proangiogenic TAMs, tissue-resident macrophage-like TAMs, and proliferating TAMs suggesting that macrophage classification is more nuanced than the binary classification [4, 5].

High infiltration of TAMs is usually associated with poor prognosis in several cancer types due to their predisposition towards an M2-like function [6]. Therefore, the M1/M2 macrophage ratio has become an essential determinant of antitumor immunity [7]. Due to their plastic nature, strategies to diminish M2-like macrophages or enhance M1-like macrophages within the TME have gained prominence [8]. The anticipated outcome of such strategies is effectively increasing the M1/M2 ratio, where a higher M1/M2 ratio is a positive indicator in immunotherapies [9].

Macrophage-based adoptive cell therapies have been tried earlier with less success owing to the plasticity of macrophages, where transplanted macrophages polarize into tumor-supporting M2-like macrophages post-transplantation [10]. Recently, histone deacetylase (HDACs) inhibitors such as HDAC6 inhibitors (HDAC6is) have demonstrated immunomodulatory effects, particularly on macrophages [11–14]. In this study, to effectively reprogram macrophages toward a proinflammatory M1 phenotype, we treated M1 macrophages ex-vivo with HDAC6i followed by intratumor adoptive cell therapy (ACT). Post-ACT, macrophages were viable, retained the M1 phenotype, and enhanced antitumor immunity by increasing the infiltration of CD8 effector T-cells into the TME. Moreover, ACT increased the macrophage M1/M2 ratio by polarizing the host tumor macrophages towards the M1 phenotype. Ex vivo inhibition of HDAC6 in M1 macrophages attenuated STAT3-mediated M2 polarization in the immunosuppressive TME, thereby sustaining the M1 phenotype post-transplantation. Single-cell transcriptomics analysis validated the ACT-mediated M1/M2 ratio increase observed by flow cytometry and immunohistochemistry. Additionally, computational analysis of cell-cell interactions and trajectory of tumor monocytes indicated that transplanted macrophages have an immune-activating effect on the TME. We validated the tumor suppressive effect of macrophage ACT in immunocompetent models, SM1 syngeneic murine melanoma, and humanized NSG-SGM3 mice with melanoma patient-derived xenograft (PDX) tumors. Our study further validates the immunomodulatory effects of HDAC6 inhibitors and demonstrates the effectiveness of macrophage-based cell therapy in treating solid tumors.

## Results

### A higher M1/M2 macrophage ratio indicates antitumor immunity in melanoma

**Fig. 1:**
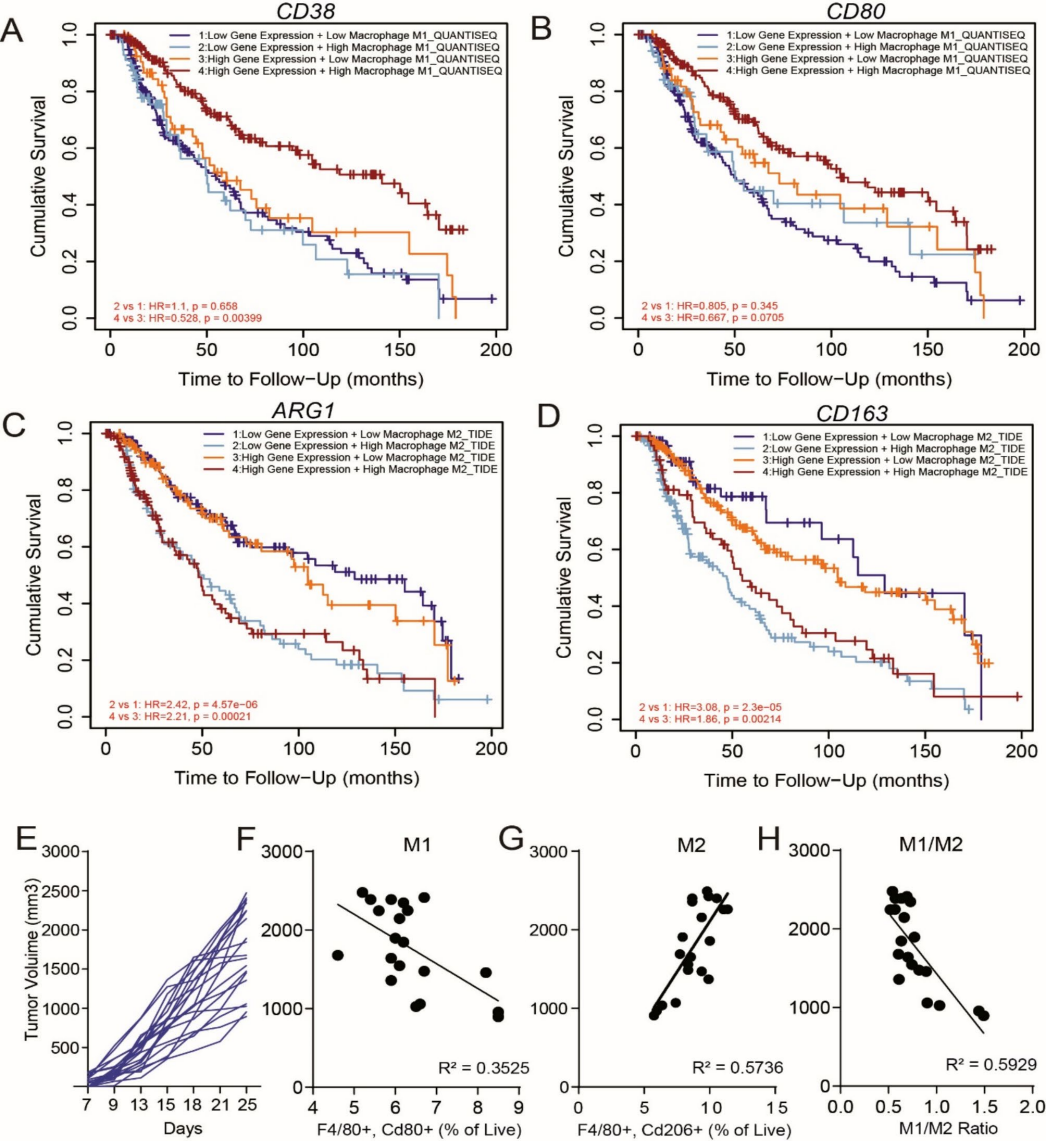
M1/M2 ratio reflects the immune status of the tumor microenvironment. (**A**-**D**) Kaplan Meier survival analysis of key M1 macrophage genes *CD38*, *CD80*, and M2 macrophage markers *ARG1*, *CD163* with overall survival in the cancer genome atlas (TCGA) skin cutaneous melanoma patients (SKCM) (*n* = 470). High or low gene expression of macrophage phenotype markers is correlated to increased or decreased presence of tumor associated macrophages (**E**) Tumor growth chart representing the growth kinetics of SM1 murine melanoma tumors with *Braf*V600E mutation in immunocompetent C57BL/6 mice at the tumor volume endpoint which was 25 days post-tumor implantation (20 mm diameter). (*n* = 20 mice) (**F**) Negative correlation between SM1 murine melanoma tumor volume and tumor-associated M1 macrophages (F4/80 + Cd80 + as % of live cells). (**G**) Positive correlation between the tumor volume and tumor-associated M2 macrophages (F4/80 + Cd206 + as % of live cells). (**H**) Negative correlation between the tumor volume and M1/M2 macrophage ratio

TAMs play a critical role in shaping the immune status of tumors. Responding to the cues from different TME cells, TAMs exhibit a broad spectrum of phenotypes, ranging between M1 and M2 macrophages or even adopting hybrid phenotypes [15]. To understand the role of macrophages in melanoma TME, we first evaluated the composition of M1-like and M2-like macrophages in human and murine melanoma tumors. High expression of M1 markers such as *CD38* and *CD80* and high infiltration of M1-like macrophages were associated with improved overall survival in the TCGA SKCM dataset (Fig. 1A-B), whereas high expression of M2 markers *ARG1* and *CD163* and high M2-like macrophage tumor infiltration was associated with poor survival (Fig. 1C-D). We further validated the role of macrophages in C57BL/6 mice engrafted with SM1 melanoma tumors and evaluated for correlation between macrophage phenotypes and tumor growth. At the endpoint, 25 days post-tumor implantation, we assessed the infiltration of M1 (F4/80+, Cd80+) and M2 macrophages (F4/80+, Cd206+), plotted as a percentage of total live cells. Figure 1E represented the tumor growth kinetics, and Fig. 1F indicated that antitumor M1 macrophages negatively correlated with tumor volume, whereas protumor M2 macrophages positively correlated with tumor volume (Fig. 1G). Figure 1H revealed a negative correlation between the M1/M2 ratio and tumor growth, which has also been observed in clinical studies [16], suggesting that the SM1 murine melanoma model is ideal for investigating the role of macrophages in tumor progression. Overall, our data indicate that TAMs polarized towards M2 phenotype play a critical role in the progression of both human and murine melanoma tumors.

### HDAC6 inhibition affects macrophage phenotype and function both in vitro and in vivo

**Fig. 2:**
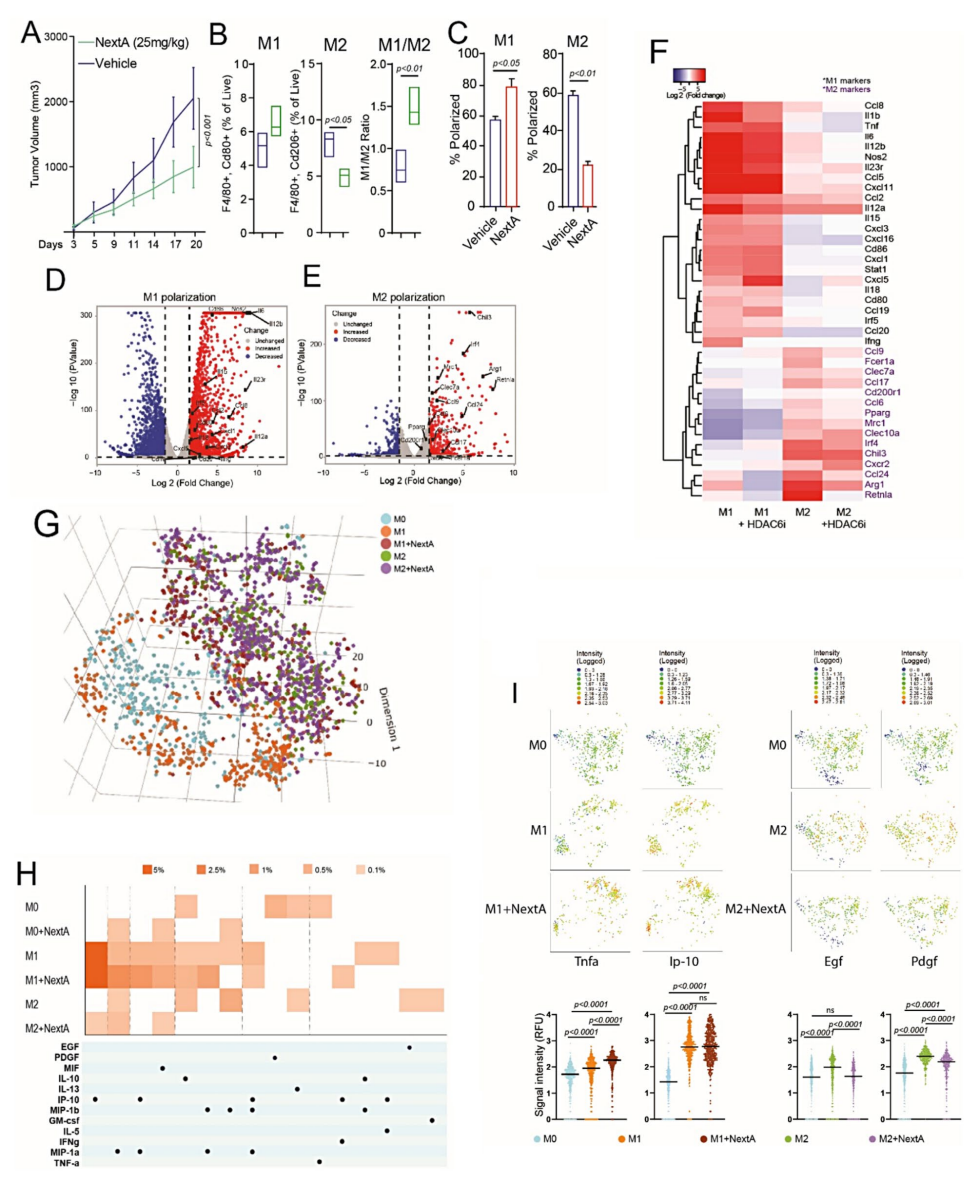
HDAC6 inhibition regulated M2 macrophage phenotype. (**A**) Tumor growth chart of syngeneic SM1 murine melanoma tumors in C57BL/6 mice treated with intraperitoneal administration of 25 mg/kg HDAC6 inhibitor, NexturastatA (NextA) or vehicle. (**B**) Tumor-associated M1 (F480 + Cd80+) and M2 (F4/80 + Cd206+) macrophages as a % of live cells, respectively, and M1/M2 ratio in vehicle or NextA treated mice bearing SM1 murine melanoma tumors. Tumors were collected on day 20 with the tumor size endpoint as 20 mm diameter. (**C**) Polarization efficiency of murine bone marrow-derived macrophages (BMDMs) to M1 and M2 phenotypes after treatment with HDAC6i, NextA (5µM) determined by flow cytometry. (**D**) Volcano plot showing fold-change and p-value for comparing vehicle-treated M1 versus M0. M0 are naïve macrophages derived from the mouse bone marrow. (**E**) Volcano plot showing fold-change and p-value for the comparisons and vehicle-treated M2 versus M0 macrophages. The significance level was determined by log2 fold changes ≥ 1.5 (upregulation/increased) or ≤-1.5 (downregulation/decreased) and p-value < 0.05. Differentially expressed genes are depicted in blue and red, where M1 and M2 markers are labeled in figures (**D**) and (**E**), respectively. (**F**) Heatmap of known markers for classically activated M1 and M2-like macrophages. Differential expression for HDAC6 inhibition versus vehicle was performed, and M1 markers (black) and M2 markers (purple) were represented using the log 2 transformed fold changes relative to vehicle-treated M0. (**G**) We analyzed about 659 M0, 407 M1, and 450 M2 macrophages treated with vehicle; 424 M1 and 378 M2 macrophages treated with NextA on mouse innate immune IsoCode chips on the Isoplexis platform. Uniform manifold approximation and projection (UMAP) dimensionality reduction analysis of BMDMs at a single cell resolution separated them into defined clusters based on their secretome profile. (**H**) A polyfunctionality heatmap representing each macrophage phenotype secretes more than one cytokine/chemokine. The intensity of orange squares in the heatmap represents the percentage of cells secreting the cytokine/chemokine indicated by corresponding black dots below. (I) 2-D tsne plots where each cell is represented as a colored dot. As shown in the intensity scale, blue indicates low expression, and red indicates high expression. Bar graphs of respective cytokines are represented as signal intensity. Proinflammatory cytokines Tnfa, and T-cell recruiting chemokine Ip-10 (Cxcl10) are elevated in M1 + NextA, whereas growth factors Egf and Pdgf secreted by M2 phenotype are decreased with NextA treatment

Previous studies proved that HDAC6i treatment reduced tumor growth and is associated with an enhanced M1/M2 ratio [11, 12, 14]. In the SM1 murine melanoma model, intraperitoneal administration of the HDAC6i NexturastatA (NextA) resulted in reduced tumor size compared to the vehicle-treated group (Fig. 2A). Phenotyping of TAMs by flow cytometry showed a slight increase in M1 macrophages and a significant decrease in M2 macrophages, thus effectively increasing the M1/M2 ratio in the NextA treated group (Fig. 2B). Similarly, BMDMs treated with NextA indicated an increase in M1 polarization and significant reduction of M2 polarization, consistent with in-vivo observations (Fig. 2C).

Transcriptional regulation of macrophage phenotypic markers by HDAC6i has not yet been reported, so we performed bulk RNA-seq analysis from BMDMs, including M0, M1, and M2 macrophages treated with vehicle or NextA. Volcano plot of gene expression changes in Fig. 2D represented an increased expression of M1 markers and proinflammatory genes, including *Nos2*, *Cd86*, *Il12a*, *Il12b*, *Ifng*, *Cxcl1*, *Cxcl3*, and *Il6*. On the other hand, M2 polarization upregulated M2 markers and anti-inflammatory genes in Fig. 2E, including *Arg1*, *Retnla*, *Mrc1*, *Chil3*, *Ccl17*, *Ccl24*, and *Irf4*. Thus, transcriptomic analysis confirmed that gene expression profiles of polarized macrophages correlated with previously reported studies [17]. The bar plot shows an exclusive expression of M1-specific genes (Supp. Fig. S1A) and M2-specific genes (Supp. Fig. S1B) in respective phenotypes, represented as heatmaps in Supp. Fig. S1C. Unsupervised cluster analysis of phenotypic markers between vehicle and NextA-treated M1 and M2 macrophages indicated two major clusters in the heatmap, as shown in Fig. 2F. One cluster included proinflammatory M1 genes wherein expression was maintained or slightly increased with NextA. In the other cluster, expression of M2 markers such as *Retnla*, *Arg1*, and *Mrc1* was decreased with NextA, suggesting a global suppression of M2-associated gene signature. Further, pathway analysis of differentially expressed genes indicated a significant downregulation of anti-inflammatory cytokine IL10 signaling with NextA treatment of M1 macrophages (Suppl. Fig. S1D), potentially enhancing the proinflammatory nature of M1 macrophages. In M2 macrophages, NextA treatment suppressed several cell cycle and cell proliferation-related pathways (Suppl. Fig. S1F).

To validate the functional consequence of HDAC6 inhibition on macrophage function, we performed a single-cell secretome (sc-secretome) analysis of BMDMs treated with vehicle or NextA. UMAP analysis of single cells in Fig. 2G separated macrophages into distinct clusters based on their secretome profiles. Figure 2H is a polyfunctional heatmap of macrophages capable of secreting more than one cytokine or chemokine, indicating the percentage of M1 and M1 + NextA macrophages secreting chemokine Ip-10 (Cxcl10), Mip-1a (Ccl3), Mip-1b (Ccl4), and Mif. In M2 macrophages, NextA reduced the percentage of macrophages secreting growth factor Egf and anti-inflammatory cytokines Il-10 and Il-13 while increasing the percentages of M2 macrophages secreting Ip-10, Mip-1a, and Mif, suggesting a shift away from M2 function. 2D-tsne plots indicated clusters of cells with increased expression of inflammatory cytokines Tnfa and T-cell recruiting Ip-10/Cxcl10 in M1 + NextA compared to M1 or M0 macrophages (Fig. 2I). Conversely, NextA treatment in M2 macrophages suppressed M2 function by decreasing the secretion of growth factors such as Egf and Pdgf compared to M2 macrophages. Thus far, the data from in-vivo study, transcriptomic analysis, and sc-secretome analysis of BMDMs comprehensively demonstrated that HDAC6 inhibition profoundly affected the macrophage phenotype and function.

### HDAC6 inhibition predominantly affects M2 macrophages

**Fig. 3:**
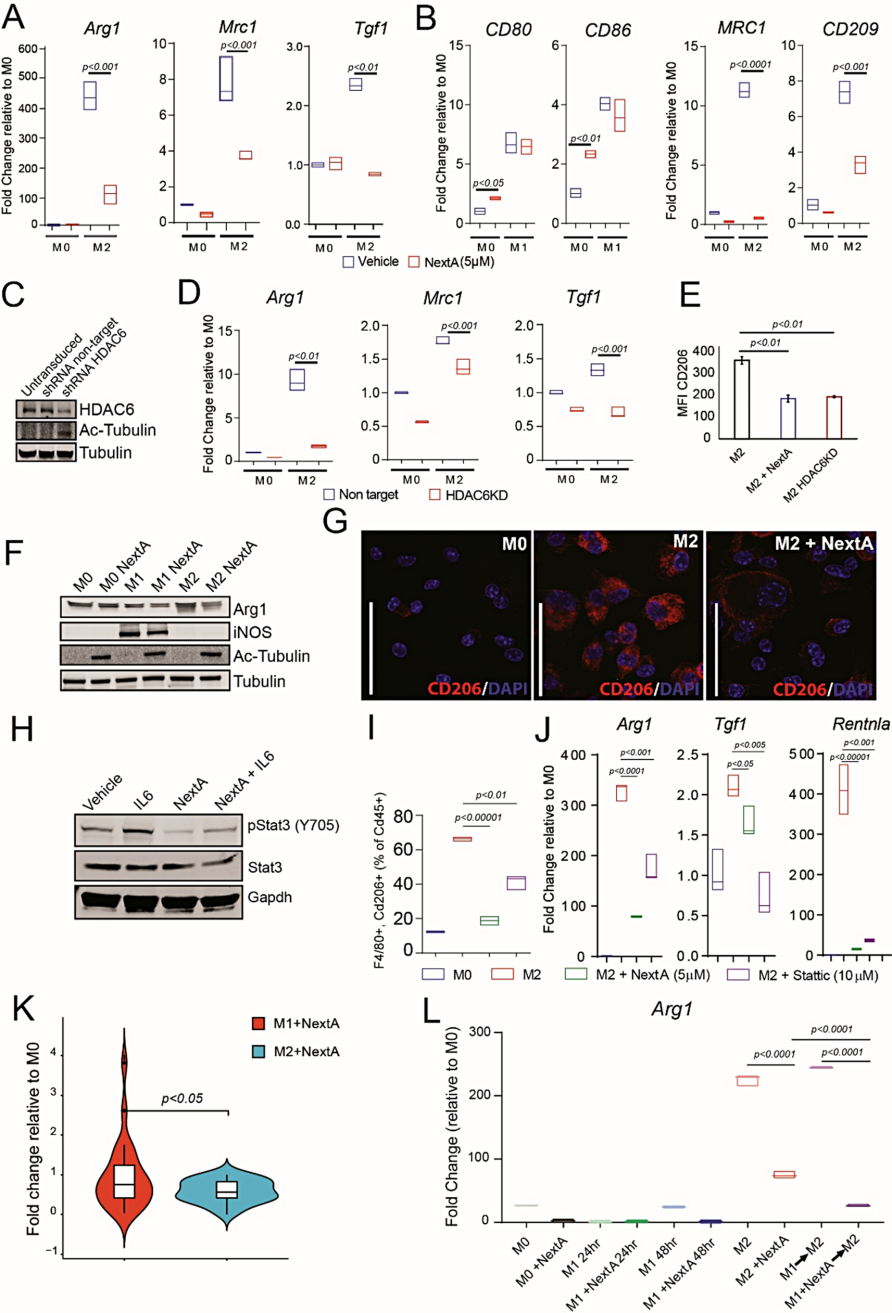
HDAC6 inhibition suppressed macrophage polarization towards the M2 phenotype. (**A**) Real time quantitative PCR (qRT-PCR) analysis of mRNA expression levels of M2 markers *Arg1*, *Tgfb1*, and *Mrc1* in vehicle and NextA (5µM) treated murine BMDMs. (**B**) Analysis of mRNA expression levels of M2 markers *MRC1* (CD206) and *CD209*, M1 markers *CD80* and *CD86* in vehicle and NextA (5µM) treated human macrophages derived from Thp1 monocytic cell line. (**C**) Immunoblot analysis of Hdac6 after non-target or *Hdac6* targeted shRNA mediated knockdown in murine macrophage cell line BMA3.1A7. Tubulin is protein loading control. (**D**) Analysis of mRNA expression levels of M2 markers *Arg1*, *Mrc1*, and *Tgf1* by qRT-PCR in non-target and *Hdac6* knockdown (HDAC6KD) BMA3.1A7 cells by q-PCR. (**E**) Mean fluorescence intensity (MFI) of CD206 in HDAC6 knock down (HDAC6KD) BMA3.1A7 murine M2 macrophages analyzed by flow cytometry. (**F**) Immunoblot analysis of BMDM macrophages for M1 (iNOS) and M2 (Arg1) markers. Tubulin is a loading control, and acetyl-tubulin is a marker for HDAC6 inhibition. (**G**) Immunofluorescence analysis of M2 marker CD206 in naïve (M0) and M2 polarized BMDMs with or without NextA treatment (5µM). (**H**) Immunoblot analysis of IL6 mediated STAT3 phosphorylation at tyrosine 705 (Y705) in RAW264.7 murine macrophages treated with NextA (5µM) or vehicle. (**I**) Flow cytometry analysis of BMDM derived M2 macrophages as a percentage of Cd45 + cells treated with HDAC6 inhibitor, NextA (5µM) and STAT3 inhibitor, Stattic (10µM). (**J**) Analysis of mRNA expression of *Arg1*, *Tfgb1*, *Retnla* (*Fizz1*) by qRT-PCR in bone marrow-derived M2 macrophages treated with NextA (5µM) and Stattic (10µM). (**K**) Violin plot representing the expression of STAT3 target genes in M1 and M2 macrophages treated with NextA shown as fold change relative to M0. Data obtained from transcriptomics analysis of murine BMDMs. (**L**) Macrophage repolarization assay, mRNA expression analysis of *Arg1* by qRT-PCR in M1 macrophages exposed to M2 polarizing cytokines

TAMs predominantly exhibit M2 phenotype [18]. Therefore, suppressing M2 macrophages could benefit cancer patients. Towards this approach, we investigated how HDAC6i could modulate M2 macrophage phenotype and function. Gene expression analysis of M2 markers indicated a significant decrease in the expression of *Arg1*,* Mrc1*, and *Tgf1* (Fig. 3A) in NextA-treated BMDMs, further validating the transcriptomics data. Of note, NextA did not induce cytotoxicity on murine BMDMs even at concentrations as high as 10µM (Supp. Fig. S2A). Therefore, the suppression of tumor growth in vivo was predominantly due to the immunomodulatory effects of NextA on the TME. Importantly, other HDAC6is have been shown to have minimal cytotoxic effects in normal and transformed cells [14].

Next, Thp1-derived human macrophages were treated with vehicle or NextA before being polarized to M1 or M2. As shown in Fig. 3B by qRT-PCR analysis, NextA significantly decreased the expression of M2 markers, *MRC1* (CD206), and *CD209* but minimally affected the polarization of M1 macrophages (*CD80* and *CD86)*. A similar reduction of *CD206* expression was observed with other HDAC6is tubacin and tubastatin A (Supp. Fig. S2B). In addition to pharmacological inhibition, we used a genetic approach with shRNA to knockdown *Hdac6* in murine BMA3.1A7 macrophages. Immunoblot analysis indicated partial knockdown of Hdac6 protein in BMA3.1A7 cells (Fig. 3C), as shown by increased acetyl-tubulin, a known substrate to be deacetylated by HDAC6 [19]. Gene expression analysis of the M2 markers, *Arg1*, *Mrc1*, and *Tgf1* by qRT-PCR revealed a reduction in the expression in *Hdac6* knockdown (HDAC6KD) compared to non-target macrophages (Fig. 3D). Flow cytometry analysis indicated a decrease in M2 polarization which was comparable to NextA treatment (Fig. 3E). Evaluation of NextA-treated macrophages by immunoblot analysis of polarization markers, indicated a decrease in arginase 1 (Arg1) in M2 macrophages and no effect on induced nitric oxide synthase (iNOS) in M1 macrophages (Fig. 3F). Furthermore, analysis of M2 macrophages by immunofluorescence demonstrated a significant decrease in the M2 marker, Cd206 compared to vehicle treatment (Fig. 3G). Taken together, pharmacological or genetic inhibition of HDAC6 in both human and murine macrophages significantly decreased M2 polarization, demonstrating that HDAC6 inhibition has a substantial immunomodulatory effect on macrophages.

We and other groups previously established that HDAC6 interacts with STAT3 to regulate the expression of STAT3 target genes [20–22]. Moreover, STAT3 is reported to regulate the expression of *Arg1* in myeloid-derived suppressor cells [23]. Therefore, we reasoned that STAT3, a key transcription factor promoting the M2-like phenotype, might regulate *Arg1* expression in macrophages. Immunoblot analysis of RAW264.7 macrophages treated with IL-6 cytokine indicated STAT3 phosphorylation at tyrosine-705 (Y705), and NextA treatment decreased STAT3-Y705 phosphorylation, which aligned with our previous report [20] (Fig. 3H). Flow cytometry analysis of M2-polarized BMDMs treated with NextA or the STAT3 inhibitor, Stattic, showed a significant decrease in M2 macrophages (Fig. 3I). The expression of M2 markers, including *Arg1*, *Tgf1*, and *Retnla* (*Fizz1*) in BMDMs was significantly decreased in M2 macrophages treated with NextA (5µM) and Stattic (10µM) (Fig. 3J). Interestingly, NextA significantly affected *Arg1* and *Retnla* (*Fizz1*) gene expression more than STAT3 inhibition. As shown in Suppl. Fig. S2C, the expression of immunosuppressive cytokine *Il10* upregulated in M1 macrophages as negative feedback to the inflammatory response mediated by IFNγ and LPS was decreased with NextA treatment. Furthermore, *Socs3* expression, a negative regulator of STAT3 signaling, was upregulated in the NextA-treated M1 macrophages, which explains the downregulation of *Il10* expression. Further analysis of STAT3 target genes in M1 and M2 macrophages from RNA-seq analysis indicated that STAT3 targets in M2 macrophages were significantly suppressed than STAT3 targets in M1 macrophages (Fig. 3K). ). In addition, gene set enrichment (GSEA) analysis of M2 and M2 + NextA transcriptomes indicated a significant number of genes in the IL6-JAK-STAT3 pathway were downregulated (Suppl. Fig. S3A). STAT3 target genes in M1 and M2 macrophages treated with NextA are shown in Suppl. Table. S5. For the first time, we report that HDAC6 inhibition can affect M2 macrophage polarization through STAT3 pathway suppression.

We further performed a repolarization assay where M1-like macrophages were treated with vehicle or NextA and subsequently exposed to M2-polarizing cytokines Il-4 and Il-13. M2 polarization was demonstrated by increased M2 marker expression, *Arg1* by qRT-PCR (Fig. 3L, compare M0 vs. M2). Consistently, NextA diminished M2 polarization (Fig. 3L, compare M0 vs. M2 + NextA). Upon repolarization with M2 cytokines, *Arg1* expression significantly increased in vehicle-treated M1 macrophages but not in the NextA-treated M1 macrophages (Fig. 3L, compare M0 vs. M1→M2 vs. M1 + NextA→M2), suggesting that M1 macrophages treated with NextA exhibit a lock into the M1 phenotype. A similar effect was demonstrated using RAW 264.7 macrophages where expression of the M2 marker, *Mrc1* was suppressed in M1 + NextA macrophages upon repolarization with M2 cytokines compared to M1-like macrophages (Suppl. Fig. S2D). Taken together, either pharmacological or genetic inhibition of HDAC6 in human and murine macrophages decreased polarization of macrophages towards tumor-promoting M2 phenotype while retaining the M1 phenotype, suggesting that HDAC6is could be used as therapeutic immunomodulatory agents to control the macrophage phenotype and function.

### HDAC6 inhibitor treated macrophage-based adoptive cell therapy improved antitumor immune response in melanoma

**Fig. 4:**
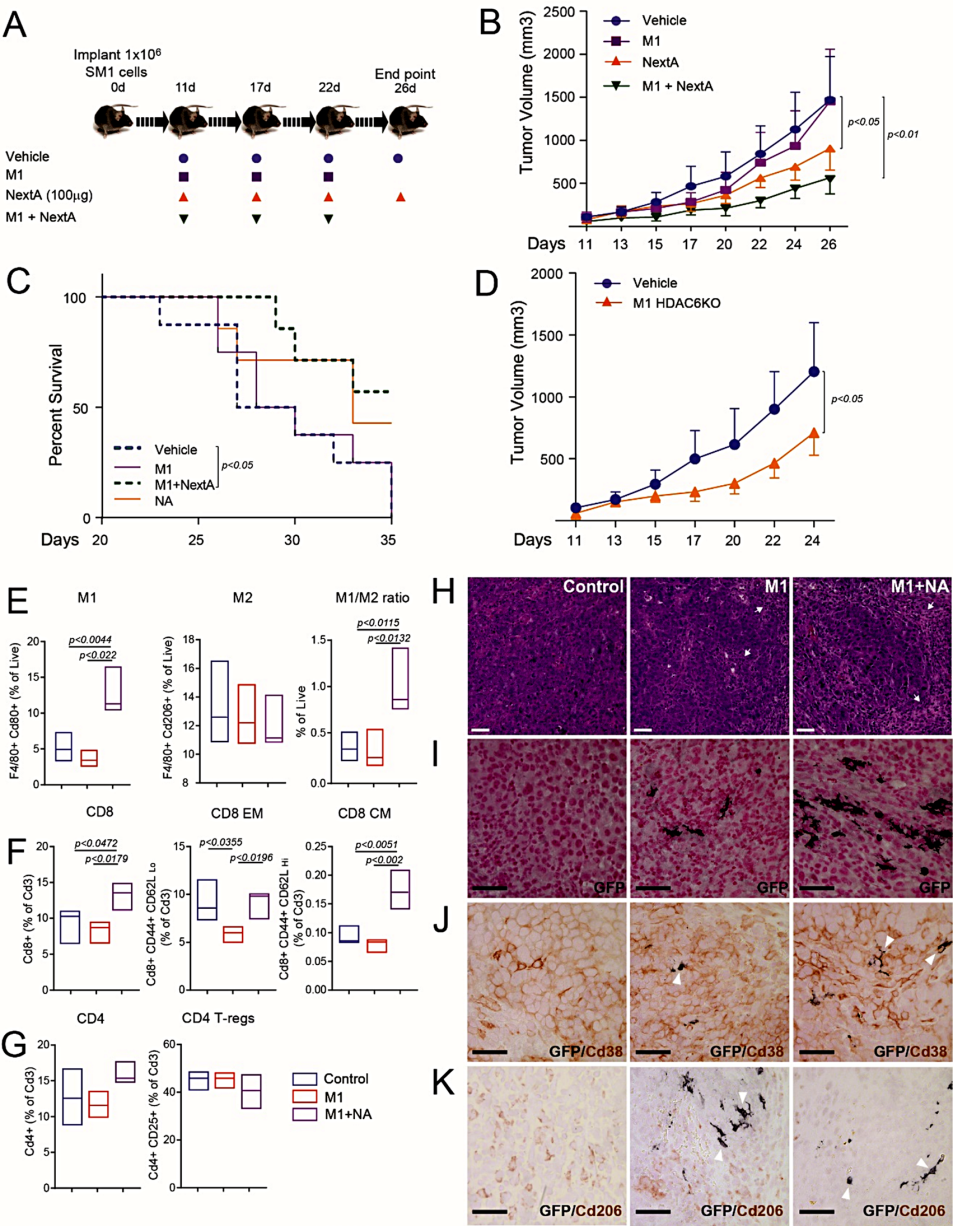
Adoptive cell therapy of HDAC6 inhibitor treated M1 macrophages diminishes melanoma tumor growth in immunocompetent mice. (**A**) Schematic workflow of the macrophage adoptive cell therapy (ACT) in C57BL/6 syngeneic SM1 murine melanoma model. (*n* = 10 mice/group) (**B**) Tumor growth chart of SM1 melanoma tumors treated with vehicle (PBS), intratumor ACT of M1 (1 × 10^6^) macrophages, injection of NextA (100ug), and intratumor ACT of M1 macrophages (1 × 10^6^) pretreated with NextA (5µM) ex-vivo. (**C**) Survival analysis of mice treated with macrophage ACT. (**D**) Tumor growth chart of SM1 murine melanoma tumors with vehicle (PBS) or bone marrow-derived M1 macrophages from HDAC6KO mouse. (**E**) Flow cytometry-based immunophenotyping of M1, M2 macrophages as a fraction of F480 + Cd80 + and F4/80 + Cd206 + macrophages, respectively, and M1/M2 ratio in SM1 murine melanoma tumors treated with macrophage ACT. (**F**) CD8 T-cells as a fraction of Cd3 + cells. (**G**) CD4 T-cells, and T-regs as a fraction of Cd3 + cells. (**H**) Analysis of SM1 murine melanoma tumors treated with vehicle (Control), M1 macrophages, or HDAC6 inhibitor treated M1 macrophages (M1 + NextA) by hematoxylin and eosin (H&E) staining. Tumor stroma is indicated with white arrows. (**I**) Transplanted macrophages were derived from the bone marrow of UBC-GFP mice with ubiquitous GFP expression, enabling us to detect them by immunostaining with an anti-GFP antibody. (**J**-**K**) Immunohistochemistry staining to detect GFP-expressing macrophages, GFP and Cd38 expressing macrophages, GFP and Cd206 expressing M2 macrophages. White arrowheads indicate transplanted GFP macrophages. GFP expression is shown in black color. Cd38 and Cd206 expression are represented in brown color

HDAC6 inhibition in macrophages decreased the M2 phenotype while retaining the M1 phenotype, offering an ideal adoptive cell therapy strategy (ACT). Therefore, we tested this approach with BMDMs treated ex-vivo with NextA and then transplanted into SM1 tumors. ACT is shown in the schematic in Suppl. Fig. S2E where macrophage ACT only resulted in a significant tumor growth reduction with NextA-treated M1 macrophages but not with vehicle-treated M1 macrophages. M0 and M2 macrophage transplantation with NextA did not reduce the tumor size significantly. Encouraged by the tumor reduction with HDAC6-inhibited M1 macrophages, we expanded our macrophage ACT to a larger cohort of mice at time points shown in the schematic in Fig. 4A. Intratumor dose titration studies indicated that 5 mg/kg of NextA (Suppl. Fig. S2F), and 1 × 10^6^ M1 + NextA treated macrophages (data not shown) were optimal to elicit a tumor reduction response. Furthermore, a cytotoxicity assay performed with NextA indicated minimal toxicity at 5µM compared to Panobinostat (LBH-589), which is a pan-HDAC inhibitor inducing significant cytotoxicity at comparable NextA concentration (Suppl. Fig. S2G). Therefore, any potential tumor reduction from NextA intratumor administration is due to tumor immune modulation rather than a direct cytotoxic effect. Consistent with Suppl. Fig. S2E, intra-tumor ACT of NextA-treated M1 macrophages resulted in a significant tumor size reduction compared to other treatment groups (Fig. 4B). NextA treatment performed better than vehicle-treated M1 macrophage ACT and the control group. The tumor reduction also translated into better survival with M1 + NextA followed by NextA and M1 groups, as shown by the Kaplan-Meier survival plot in Fig. 4C. More importantly, we also observed that ACT with BMDMs derived from HDAC6 knockout mouse recapitulated the results of M1 + NextA (Fig. 4D). Overall, ACT with NextA-treated M1 macrophages significantly reduced tumor volume and improved survival compared to the control group, highlighting the efficacy of macrophage ACT.

Flow cytometry analysis indicated a sharp increase in M1 macrophages and no change in M2 macrophages in the M1 + NextA ACT group compared to Vehicle and M1 macrophage ACT treatment groups. This increase in the M1 macrophages from ACT translated into a higher M1/M2 ratio in the M1 + NextA group. (Fig. 4E). The Higher M1/M2 ratio was further associated with an increased infiltration of CD8 + effector T-cells in the M1 + NextA group (Fig. 4F). There was also an increase in effector memory (EM) cells and central memory (CM) CD8 T-cells. Despite a trend towards an increase in CD4 + cells, there was no significant difference in the immunosuppressive regulatory T-cells (T-regs) among the treatment groups (Fig. 4G). Overall, an increase in the number of pro-inflammatory immune cells, including M1 macrophages and CD8 effector T-cells, suggested that macrophage ACT activated both innate and adaptive antitumor immunity.

Histological analysis of tumor sections from vehicle, M1, and M1 + NextA treatment groups (Fig. 4H) showed areas of increased stroma (white arrows) in the M1 and M1 + NextA tumor sections compared to the vehicle-treated group, suggesting there may be an overall increase in the infiltration of immune cells. Notably, GFP-positive M1 and M1 + NextA macrophages (stained black) were still viable two weeks post-transplantation (Fig. 4I). Further analysis of M1 marker Cd38 (stained brown) visibly indicated an increased presence of M1 macrophages in the proximity of GFP macrophages in both M1 and M1 + NextA groups. Importantly, some GFP macrophages stained for both GFP and Cd38 (white arrowheads), demonstrating that transplanted M1 + NextA macrophages retained the M1 phenotype (Fig. 4J). On the other hand, M2 tumor macrophages were reduced in the vicinity of M1 GFP macrophages (as indicated by white arrowheads) treated with NextA, suggesting a proinflammatory TME in the M1 + NextA group (Fig. 4K). Overall, HDAC6 inhibition in ACT macrophages significantly reduced tumor volume through antitumor M1 macrophages and increased infiltration of CD8 effector T-cells.

### Single-cell profiling of tumor immune infiltrate reveals a proinflammatory TME post macrophage ACT

**Fig. 5:**
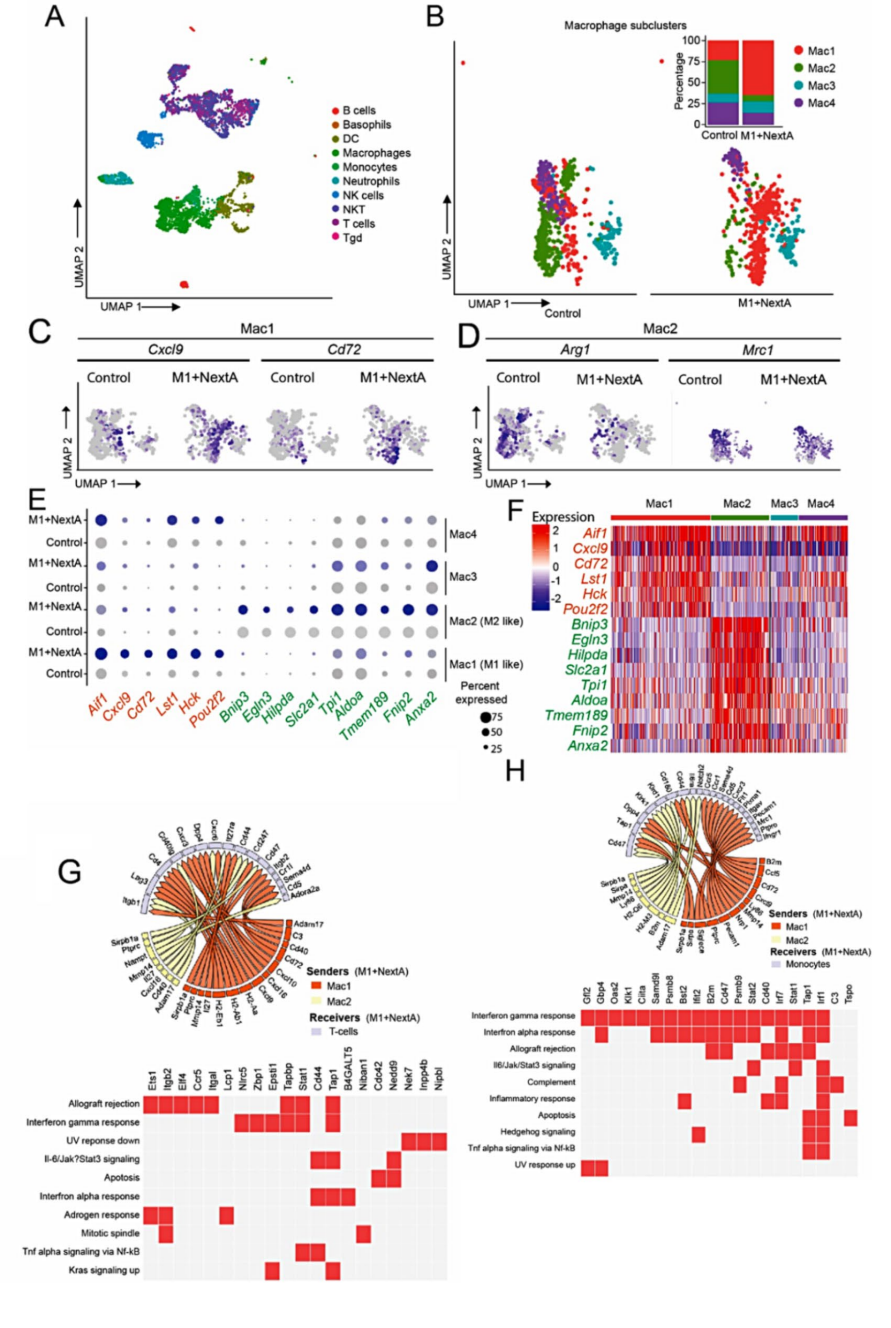
Macrophage ACT activated inflammatory immune responses in the TME. (**A**) UMAP analysis of Cd45 + sorted tumor infiltrated immune cells from Control and M1 + NextA macrophage treated tumors analyzed with 10xGenomics workflow. UMAP cluster analysis of aggregated immune cells by SingleR cell annotation revealed major immune cell populations. (**B**) Tumor macrophage subclusters Mac1, Mac2, Mac3, and Mac4 show differential presence represented as a percentage of total tumor macrophages (insert with stacked bar plot). (**C**) Feature plots representing the expression of inflammatory genes *Cxcl9* and *Cd72* associated with M1 phenotype in Mac1 macrophage subcluster in Control and M1 + NextA tumors. (**D**) Feature plots representing expression of tumor promoting genes *Arg1* and *Mrc1* associated with M2 phenotype in Mac2 macrophage subcluster in Control and M1 + NextA tumors. (**E**) Bubble plot showing differentially expressed top significant gene between macrophage subcluster. Mac1 associated genes are shown in orange, and Mac2 associated genes are shown in green color. (**F**) Heatmap representing the expression of Mac1 and Mac2 gene signatures in single cells across 4 macrophage subclusters. (**G**) Circos plot showing potential interactions between ligands expressed by macrophages in Mac1 and Mac2 subclusters and receptors expressed on the receiver T-cells. Heatmap representing the pathway analysis of activated target genes in receiver monocytes. MSigDB Hallmark 2020 module in Enrichr was used to generate the heatmap. (**H**) Circos plot showing potential interactions between ligands expressed by macrophages in Mac1 and Mac2 subclusters and receptors expressed on the receiver monocytes and corresponding heatmap of pathway analysis

scRNA-seq analysis of myeloid cells across multiple cancers revealed the diversity of TAMs ranging from inflammatory-proliferating to regulatory TAMs [4]. Therefore, we explored the cellular composition of SM1 tumors post-ACT using the 10X Genomics platform. UMAP analysis [24] of sorted Cd45 + cells isolated from tumors indicated distinctive clusters of immune cells from Control and M1 + NextA tumors. SingleR analysis [25] revealed major clusters including macrophages, monocytes, and T-cells (Fig. 5A). Further analysis of macrophages resolved into four distinct subclusters Mac1, Mac2, Mac3, and Mac4 (Fig. 5B). The control tumor was predominant with Mac2 and Mac4 subclusters; whereas the M1 + NextA tumor group was enriched with Mac1 subcluster. Gene expression analysis indicated the Mac1 subcluster expressed an inflammatory gene signature and a Mac2 subcluster expressed an anti-inflammatory signature akin to M1 and M2 macrophages, respectively. Therefore, ACT with M1 + NextA macrophages increased the percentage of M1-like macrophages with a concomitant decrease of M2-like macrophages (Fig. 5B **insert**). Feature plots of M1-like genes *Cxcl9*, *Cd72* (Fig. 5C), *CD80*, and *CD86* (Suppl Fig. S4A) indicate expression in the Mac1 subcluster of M1 + NextA tumors. Conversely, M2 genes, *Arg1* and *Mrc1* (Fig. 5D) and Thbs1 (Suppl Fig. S4B) were mostly expressed by the Mac2 subcluster in the control tumors. Differential gene expression analysis of macrophage subclusters enriched for *Aif1*, *Cxcl9*, *Cd72*, *Lst1*, *Hck*, and *Pou2f2* gene signature in the Mac1 that are linked to macrophage activation, phagocytosis, T-cell recruitment and inflammatory response [26], [27]. Overall, the Mac1 subcluster was similar to the inflammatory TAMs reported by Ma et al. [4]. Genes enriched in the Mac2 subcluster included *Bnip3*, *Egln3*, *Hilpda*, *Slc2a1*, *Tpi1*, *Aldoa*, *Tmem189*, *Fnip2*, and *Anxa2* and its gene signature was similar to the regulatory TAMs reported by Ma et al. [4]. We observed that the Mac1 gene signature was partially expressed by the Mac4 subcluster, while the Mac2 gene signature was partially expressed by the Mac3 subcluster, as shown in the bubble plot in Fig. 5E and the heatmap in Fig. 5F. We further interrogated the expression of M1 and M2 gene signatures identified from bulk RNA-seq (Fig. 2F) and found that M1 gene signature was upregulated in the ACT tumor macrophages (Suppl Fig. S4C) and M2 gene signature was upregulated in the Control tumor macrophages (Suppl. Fig. S4D). The data thus far indicated that M1 + NextA macrophage ACT resulted in an increased M1/M2 ratio, and the expression of M1-associated gene signature is positively correlated to survival benefits to patients.

**Fig. 6:**
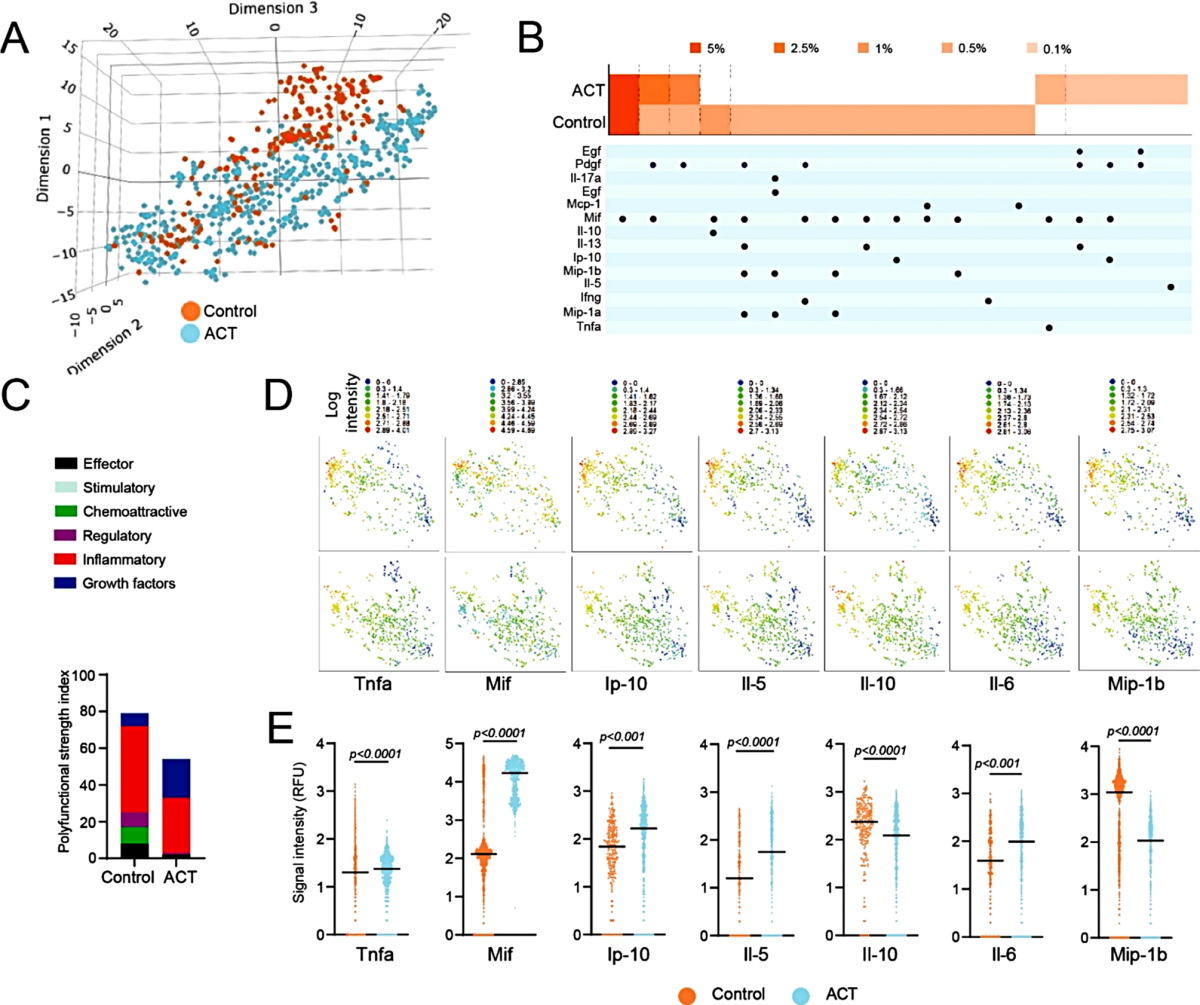
Single-cell secretome analysis indicated polyfunctionality of tumor macrophages. Two weeks post-ACT with NextA-treated M1 macrophages, tumors were harvested and flow-sorted for TAMs with Cd45 and F40/80 antibodies. About 247 TAMs from the control group and 585 TAMs from the M1 + NextA ACT group were analyzed with high IsoQ scores. (**A**) A 3D-tsne plot of Cd45 + F4/80 + sorted tumor macrophages from Control and M1 + NextA macrophage ACT treated tumors analyzed on IsoSpark. (**B**) Polyfunctionality heatmap representing the percentage of cells expressing different cytokines and chemokines by tumor macrophages from Control and M1 + NextA ACT tumors. (**C**) Polyfunctionality strength index of TAMs expressing cytokines and chemokines. (**D**-**E)** 2-D Tsne plots and scatter plots of signal intensities of represented secreted factors from TAMS isolated from Control and M1 + NextA ACT tumors

To further understand the impact of NextA-treated M1 macrophage ACT on the TME, we performed sc-secretome profiling of TAMs on the Isoplexis platform using a mouse innate immune IsoCode chips. The 3D-tsne plot indicated the clustering of TAMs from control tumors separated from ACT-treated tumors based on their secretome profiles (Fig. 6A). Compared to BMDMs, TAMs were relatively more polyfunctional, as indicated by the number of secreted cytokines and chemokines shown in heatmap, potentially due to a niche of cell-cell communication that is absent in BMDMs cultured in-vitro (Fig. 6B). The majority of TAMs from both control and ACT tumors secreted inflammatory cytokines, as shown in the polyfunctional strength index plot in Fig. 6C. Expression of cytokines and chemokines by TAMs are shown as 2-D Tsne plots in Fig. 6D. Compared to the control tumor, TAMs from the ACT tumor had increased secretion of inflammatory factors including Tnfa, Mif, T-cell recruiting chemokine Ip-10, and Il-5, whereas decreased secretion of anti-inflammatory Il-10. Pleotropic cytokines Il-6 secretion was increased, while Mip-1b (Ccl4) was reduced with ACT TAMs (Fig. 6E). Thus, the sc-secretome data indicated that ACT with NextA-treated M1 macrophages altered the function of TAMs towards an inflammatory phenotype.

### Ligand-receptor interaction analysis indicated immune activating signals upregulated with macrophage ACT niche

Cell-to-cell communication through secretory factors and ligand-receptor interactions sustains the dynamic nature of the TME. We previously reported that a functional immune system is required for HDAC6 inhibition-mediated tumor suppression using immunodeficient SCID mice and CD4/CD8 T-cell depletion assays [28]. This was particularly evident from sc-secretome analysis, where TAMs were relatively more polyfunctional compared to in vitro cultured BMDMs due to the interactive nature of the TME. Therefore, we interrogated whether macrophage ACT affected cell-cell interactions through differential NicheNet analysis. The top 30 ligand-receptor interactions were represented as a heatmap (Suppl. Fig. S5A) showing potential interactions between macrophage ligands and T-cell receptors. In the M1 + NextA niche, ligand-receptor interaction analysis indicated that Cd72 in the Mac1 subcluster potentially interacted with Sema4d and Cd5, expressed on T-cells in M1 + NextA tumors. Cd72 is expressed in a subset of inflammatory macrophages [29], while Sema4D (Cd100) [30] and Cd5 [31] are highly expressed in T cells. Similarly, T-cell recruiting Cxcl9 and Cxcl10 chemokines were upregulated in the Mac1 subcluster, potentially recruiting T-cells into M1 + NextA -treated tumors through interaction with Cxcr3. Increased expression of Cxcl10 (Ip-10) was validated by sc-secretome analysis of TAMs isolated from M1 + NextA tumors (Suppl. Fig. S4E). Next, we analyzed ligand activity based on the expression of target genes in receiver cells. Il27a and Ebi3 (Il27b) were the most active among the ligands, with the ability to activate most of the top target genes (Suppl. Fig. S5B). The circos plot in Fig. 5G summarizes potential interactions between the ligands and receptors expressed on the Mac1 and Mac2 macrophage subclusters and tumor-infiltrated T-cells. Pathway analysis of top target genes in T-cells indicated activation of allograft rejection and interferon-gamma response among the significant pathways based on p-values. Overall, NicheNet analysis demonstrated macrophage-mediated T-cell activation.

Since monocytes are the major contributors to the macrophage population in tumors, we interrogated the macrophage-monocyte interactions using NicheNet analysis. Similar to T-cells, the interaction of ligands Cd72, Cxcl9, and Cxcl10 from Mac1 was also observed in monocytes. Furthermore, monocyte recruiting chemokine Ccl5 from Mac1 potentially interacts with Ccr1 in Control tumor monocytes. Also, this interaction was upregulated with Ccl5 in M1 + NextA tumor monocytes (Suppl. Fig. S5C). In addition, Ccl12 from both Mac1 and Mac2 subclusters interact with Ccr2 in M1 + NextA tumor monocytes. Adam17, Ltb, Ptprc, Ly86, Ccl12, and Tnfsf13b appear to be the most active ligands potentially upregulating most top-scored target genes, including Stat1 transcription factor (Suppl. Fig. S5D). Pathways analysis of top target genes in monocytes reflected that interferon-gamma and interferon alpha response were among the significant pathways based on p-values. The circos plot in Fig. 5H summarizes potential interactions between the ligands and receptors expressed on the Mac1 and Mac2 macrophage subclusters and tumor-infiltrated monocytes.

In addition, we analyzed the fate of monocytes by trajectory analysis (Suppl. Fig. S6A). Macrophages in the control tumor clustered as several subsets transitioning from infiltrated monocytes. On the other hand, M1 + NextA tumor macrophages clustered exclusively into a dichotomous grouping. Pathway analysis of top 100 significant genes in Node 1 macrophages was enriched with oxidative phosphorylation, suggesting a more M2 phenotype (Suppl. Fig. S6B). Node 2 macrophages were enriched with inflammatory response pathways, which is evident by macrophage activation GO biological process demonstrating more M1-like characteristics (Suppl. Fig. S6C). The Node 3 was enriched with Il-2/Stat5 signaling pathway and Jak-Stat receptor signaling biological process representing an intermediate phenotype between M1 and M2 macrophages (Suppl. Fig. S6D). More importantly, in M1 + NextA tumors, transitioning monocytes appear closely aligned to Node 2 inflammatory M1-like macrophages compared to the control tumor macrophages. Overall, computation analyses indicated that Mac1 subcluster from differential NicheNet and Node2 macrophages from pseudotime analysis have a proinflammatory and activating effect on tumor-infiltrated T-cells and monocytes. This agrees with the biological responses such as increased M1/M2 ratio and increased CD8 T-cell infiltration with macrophage ACT in vivo studies.

Finally. we performed an in-vitro co-culture assay of T-cells and macrophages with or without NextA treatment (5µM) for 72 h to validate macrophage-mediated T-cell activation. Analysis of supernatant for cytokine and chemokine secretome indicated an increased secretion of proinflammatory factors such as IP-10 (Cxcl10), IFNg, TNFa, and RANTES by T-cell co-cultured with M1 + NextA macrophages (similar to M1 + NextA tumor macrophages) than T-cells cultured with M1 macrophages (Suppl. Fig. S6E). Furthermore, the proliferation of CD8 T-cells suppressed when co-cultured with M2 macrophages was rescued with NextA-treated M2 macrophages (Suppl. Fig. S6F), suggesting that HDAC6 inhibition can influence T-cell activity through macrophages.

### Macrophage ACT in humanized mice reduced melanoma tumor growth

Next, we used human macrophages derived from circulating monocytes to demonstrate the translational potential of macrophage-based ACT in treating cancers. Sc-secretome analysis of human M1 or M2 macrophages treated with NextA revealed well-defined clustering of macrophages in a 3D-tsne plot, suggesting that each macrophage phenotype has a unique secretome profile (Suppl. Fig. S7A). Polyfunctional heatmap indicated that NextA treatment increased the percentage of polyfunctional M1 macrophages secreting more than one cytokine or chemokine (Suppl. Fig. S7B). More red dots in 2D-tsne plots (Suppl. Fig. S7C) of M1 + NextA macrophages indicate increased secretion of proinflammatory cytokines IL-18 and GM-CSF with NextA treatment compared to M0 and M1 macrophages. However, IL-12p70 was not affected by HDAC6 inhibition. The signal intensity scatter plots indicate the level of expression of respective cytokines shown in 2D-Tsne plots Suppl. Fig. S7E. On the other hand, secretion of immunoregulatory cytokine IL-13 and soluble CD40L by M2 macrophages was not affected by NextA, whereas growth factor VEGF was significantly decreased with HDAC6 inhibition (Suppl. Fig. S7D). The signal intensities of corresponding cytokines from each cell are shown as scatter plots in (Suppl. Figs. S7E-F). Overall, inhibition of HDAC6 with NextA enhanced the inflammatory function of human M1 macrophages but also decreased the M2 function similar to murine BMDMs.

We applied a macrophage ACT approach similar to SM1 murine melanoma with human humanized NSG-SGM3 mice harboring *BRAF* V600E mutated human melanoma tumor xenografts. Before intratumor implantation, macrophages were verified for M1 polarization by qRT-PCR analysis to express M1 markers *CD80*, *CD86*, and *TNF* (Suppl. Fig. S8A). Intratumor ACT of human macrophages into PDX tumors significantly reduced tumor size, mirroring the results from the SM1 melanoma model (Suppl. Fig. S8B). Since we did not observe significant differences in the effect of vehicle-treated M1 macrophages with the SM1 murine melanoma model, we did not include the M1 group in this experimental design. Further analysis of the immune cell composition of the TME revealed an increase in antitumor M1 macrophages as a fraction of total CD45 + cells. Similar to the SM1 model, we observed an increment in the M1/M2 ratio in those tumors subjected to M1 + NextA treatment (Suppl. Fig. S8C). Despite the trends in the number of M1 and M2 macrophages not being significant due to small sample size (*n* = 5 per group), tumor-associated macrophages’ trends were similar to the SM1 model. However, an important increase in the M1/M2 ratio *(p-value*, 0.0703) suggested that NextA-treated M1 macrophages provided antitumor immunity. Histological analysis of humanized melanoma tumors was consistent with the flow cytometry data. We observed increased M1 macrophages in the vicinity of blood vessels and towards the periphery, along with scattered distribution within the tumor, suggesting an increased infiltration of proinflammatory M1 macrophages. On the contrary, the number of M2 macrophages within the tumor was substantially lower, similar to the histological analysis of SM1 tumors (Suppl. Fig. S8D). Overall, the NSG-SGM3 model with a humanized immune system recapitulated the observations in SM1 murine melanoma model supporting HDAC6 inhibitor-treated M1 macrophages as a potential cell therapy to treat solid tumors.

## Discussion

Tumor-associated macrophages play a critical role in determining the fate of tumors depending on their antitumor or protumor properties. Therefore, strategies to manipulate TAMs present a new therapeutic strategy. Earlier studies with the administration of monocyte-derived macrophages as cell therapy did not result in significant improvement in patients with advanced cancers; however, they were well tolerated with no reported toxicities [32–34]. A potential explanation for the limited success of these therapies could be the overwhelmingly immunosuppressive nature of the TME, which influences tumor-infiltrated monocytes, resident, or transplanted macrophages towards the tumor-promoting M2 phenotype. Therefore, our solution to this obstacle is to render the transplanted macrophages resistant to changing toward tumor-promoting M2 phenotype. This is particularly relevant because chimeric antigen receptor (CAR) expressing macrophages are being explored as an option for cell therapy [35]. In our methodology, macrophages were treated ex-vivo with HDAC6i, NextA, to prolong the antitumor M1 phenotype of the transplanted macrophages while activating antitumor adaptive immune responses in the TME. A significant advantage to our methodology is that HDAC6 inhibitor treatment of M1 macrophages is performed ex-vivo, thus eliminating any potential off-target effects of HDAC6 inhibitors.

TAMs exhibit transcriptional heterogeneity and remarkable plasticity by changing phenotypes between M1 and M2 in response to cues within the TME [36, 37]. TAMs can also exhibit hybrid phenotypes with varying M1 and M2 marker expression levels. This is evident in the survival analysis of the SKCM dataset where outcomes were stratified based on the level of macrophage infiltration and expression of markers for M1 and M2 macrophages (Fig. 1A-D). Therefore, the ratio between M1-like and M2-like TAMs within the TME is a critical indicator of the tumor’s immune status. Most studies indicate that a higher M1/M2 ratio negatively impacts tumor growth with a favorable clinical outcome in various cancer types [9, 38–40]. In this study, SM1 murine melanoma tumors show a clear negative correlation between tumor volume and M1/M2 (Fig. 1H), making it an ideal model for studying the impact of manipulating macrophages in the TME.

Macrophage ACT significantly reversed the ratio from M2 dominant to M1 dominant compared to the control cohort. The source for the increase in M1 macrophages in the TME of the treatment cohort can be attributed to the following possibilities: 1). Transplanted macrophages are viable and maintain the M1 phenotype; 2). Transplanted macrophages induced resident TAMs to turn into M1 phenotype; 3). Proinflammatory TME due to the transplanted macrophages recruited monocytes that differentiate into the M1 phenotype. Histological analysis indicated that transplanted macrophages were viable and retained proinflammatory M1 phenotype after two weeks post-ACT. Coupled with the observation that M1 + NextA treated macrophages were resistant to M2 polarizing cytokines, it is evident that transplanted macrophages contributed to the M1/M2 ratio increase. However, it is also very likely that transplanted M1 macrophages induced the conversion of tumor macrophages and infiltrated monocytes toward the M1 phenotype. NicheNet analysis indicated that this could be a possibility based on the presence of inflammatory gene signature of monocytes, which was further corroborated by trajectory analysis of inflammatory monocyte to M1-like macrophage transition. Histological analysis of the M1 marker CD38 showed an increase in M1 macrophages other than GFP-stained macrophages, suggesting that transplanted macrophages may have induced a proinflammatory TME, converting host-infiltrated macrophages towards the M1 phenotype. This increase in M1 macrophages in the ACT cohorts was consistent between the SM1 tumor and humanized PDX models. Regarding the humanized mouse model, we acknowledge that due to the small sample size of NSG-SGM3 mice treated with M1 + NextA ACT, the trends of M1 and M2 macrophages are not significant, but they are consistent with the SM1 murine melanoma model. On the other hand, the M1/M2 ratio in both melanoma models has a significant increase, suggesting that it is a better metric to assess the effectiveness of ACT. We recently demonstrated that a combination of radiation therapy and intratumor ACT with macrophages treated with HDAC6i, SP-2-225 resulted in diminished tumor growth and altered the M1/M2 ratio in the TME [41]. In addition, we reported an increase in the efficacy of anti-CD47 immunotherapy in combination with HDAC6i due to the regulation of the CD47/SIRPα axis by HDAC6 inhibition resulting in increased phagocytosis by macrophages [42]. Therefore, HDAC6 inhibition in macrophages appears to regulate multiple pathways that affect their phenotype and function.

A major concern with macrophage ACT is that M1 macrophages turn towards tumor-promoting M2 phenotype post-transplantation. Our strategy addressed this issue by attenuating STAT3 signaling through HDAC6 inhibition. Previously, we reported that HDAC6 interacts with STAT3, where HDAC6 forms a complex with STAT3 in regulating the expression of anti-inflammatory IL-10 cytokine [20] and immunosuppressive checkpoint molecule PD-L1 (CD274) [21]. This is evident in M2 macrophages treated with NextA resulting in the suppression of the IL6-JAK-STAT3 pathway (Suppl. Fig. S3A). Mechanistically, we have shown that HDAC6 inhibition results in increased interaction of STAT3 with phosphatase PP2A resulting in dephosphorylation of STAT3 and thereby suppressing STAT3 pathway [21]. We acknowledge that other potential mechanisms exist that have not been identified in this study owing to the dynamic nature of the TME. However, we did observe that MYC targets were substantially downregulated in M2 macrophages treated with NextA (Suppl. Fig. S3B) suggesting an alternative mechanism for HDAC6-mediated M2 suppression. It is reported that c-Myc plays a critical role in the alternative activation (M2) of human macrophages and TAMs [43]. We speculate that inhibition of HDAC6 played a role in suppressing Myc-regulated M2 programming. In addition, HDAC6 regulates Myc stability by deacetylation, where inhibition of HDAC6 may result in hyperacetylated Myc targeted for proteasomal degradation [44]. Finally, through differential NicheNet analysis of cell-cell interactions, we validated the antitumor role of Mac1 cluster in the ACT tumor niche by activating tumor-infiltrated T-cells and inflammatory monocytes. Regarding macrophage phenotypic markers, in immunohistochemistry, CD38 expression has increasingly been seen as a reliable M1 macrophage marker and CD206 as an M2 marker in solid tumors [17, 45]. With M1 macrophage transplantation, both SM1 and NSG-SGM3 models showed an increase in CD38 + cells and a decrease in CD206 + cells, further highlighting the effectiveness of macrophage ACT.

In summary, our study addressed the bottleneck in macrophage-based antitumor cell therapies: phenotypic conversion of transplanted antitumor M1 macrophages into tumor-promoting M2-like macrophages. We achieved this by using HDAC6is to lock the transplanted macrophages into a proinflammatory and antitumor M1-like phenotype. In conclusion, we provide a rationale for considering HDAC6i-treated macrophages as a novel antitumor cell therapy modality to treat solid tumors.

## Materials and methods

### Cell culture

RAW267.4 macrophage cells were cultured in complete DMEM medium supplemented with 1% non-essential amino acids (NEAA), 1% penicillin-streptomycin (PS), and 10% fetal bovine serum (FBS) and incubated in 5% CO_2_ at 37 °C. BMA3.1A7 cells were kindly donated by Dr. Kenneth Rock from the University of Massachusetts Medical School. BMA3.1A7 macrophages were cultured in complete RPMI-1640 with 10% FBS, 1% NEAA, 1% PS, and 1% L-glutamine. The cells were passed by gently scrapping them with a cell scrapper (Fisher, Cat # 08-100-240). Murine bone marrow-derived macrophages (BMDMs) and THP-1 human monocytic cells were cultured in RPMI-1640 complete medium.

### Macrophage isolation and polarization

For adoptive cell therapy (ACT), BMDMs were isolated for intratumor transplantation from C57BL/6-Tg (UBC-GFP)30Scha/J (Jax Strain #004353) (UBC-GFP) to distinguish between endogenous TAMs and transplanted macrophages. BMDMs were also isolated from wild-type C57BL/6 mice wherever necessary. Isolated bone marrow cells were cultured in RPMI-1640 complete medium as mentioned above and incubated in 5% CO_2_ at 37 °C. For isolating BMDMs, bone marrow from the femur and tibia bones was flushed, resuspended, and cultured in 10 cm culture plates with murine recombinant M-Csf (20ng/mL). On day 4, undifferentiated and floating cells were washed with PBS and replaced with fresh RPMI media. BMDMs were allowed to incubate for another day with fresh medium. On day 6, macrophages were pretreated with 5µM of HDAC6 inhibitor NextA or vehicle prior to adding M1 polarizing factors; murine recombinant interferon-gamma (IFNγ) (50ng/mL), and bacterial lipopolysaccharide (LPS) (100ng/mL) for 24 h. The macrophages were harvested, washed thoroughly with PBS, and resuspended to a cell density of 1 × 10^6^ cells in 100uL of PBS for intratumor implantation. M2 macrophages were obtained with recombinant murine cytokines IL-4 (20ng/mL) and IL-13 (20ng/mL). For the macrophage repolarization experiments, BMDMs were polarized to M1 for 24 h in the presence of vehicle or 5µM NextA as previously described. Subsequently, M1 macrophages were repolarized to M2 through stimulation with IL-4 and IL-13. These samples were evaluated 24 h after the repolarization step.

For macrophage ACT into NSG-SGM3 mice, partial HLA-matched (A*02:01 A*02:01) frozen purified human monocytes were purchased from Stemcell Technologies (Cat#70034). Monocyte-to-macrophage differentiation was performed following the manufacturer’s instructions using ImmunoCult™-SF Macrophage Medium (Stemcell, Cat # 10961) and recombinant human M-CSF (Stemcell, Cat # 78057.1). Polarization towards the M1-like phenotype was performed by using a 6-day protocol as directed by the manufacturer’s instruction, which included LPS (10ng/mL) and recombinant human IFNγ (Biolegend, Cat # 570202) (50ng/mL) for two days. M1-like macrophages were either pretreated with HDAC6 inhibitor (NextA) (5µM) or vehicle before M1 polarization. Macrophages from THP1 cells were obtained by treating them with phorbol 12-myristate 13-acetate (PMA) (100nM) to induce differentiation into macrophages. Further polarization towards M1 and M2 phenotypes was performed with respective recombinant human cytokines, as mentioned above.

### Animal studies

Animal studies were performed per the IACUC guidelines (Protocol# A354) at George Washington University. Murine melanoma tumors were established by implanting 1 × 10^6^ SM1 cells in the right flanks of 6–8 weeks-old female C57BL/6 mice. When the tumors were palpable, mice were randomized, and NextA was administered intraperitoneally at a dose of 25 mg/kg 5 times a week till the end of the study. For macrophage adoptive cell therapy, after allowing the tumors to grow to a size of approximately 200-400mm^3^, the mice were randomly assigned to the following cohorts, and all treatments were performed intratumorally: control mice were injected with PBS, mice adoptively transferred with M1-like macrophages, mice treated with intratumor injection of NextA (100 µg), and mice implanted with M1-like macrophages pretreated with HDAC6 inhibitor NextA. Tumor measurements were obtained every other day to track the tumor growth until the endpoint (2 cm diameter in any direction). Tumor volume was calculated with the formula V = (length x width^2^)/2. The mice were euthanized at the endpoint, and tumors were harvested for flow cytometry. Humanized NSG-SGM3 mice (NOD.Cg-Prkdc^scid^ Il2rg^tm1Wjl^ Tg(CMV-IL3,CSF2,KITLG)1Eav/MloySzJ, Stock No:013062) were purchased from Jax Labs. NSG-SGM3 mice expressing three different transgenes for SCF, GM-CSF, and IL-3 driven by CMV promoter to support and promote the expansion of human myeloid cells, including monocytes and macrophages were humanized at Jackson Laboratory (JAX) with stable engraftment of human CD34 + hematopoietic stem cells. Human melanoma tumors (Jax labs, PDX Skin Cancer Model # J000106560) with HLA type A*02:01 A*02:01 were engrafted into NSG-SGM3 mice by Jax labs before undergoing macrophage ACT. Tumor growth, flow cytometry analysis, and histology were performed similarly to the SM1 murine melanoma model but with human-specific antibodies. 1 × 10^6^ cells were implanted on either side of the tumor to a total number of 2 × 10^6^ macrophages. One-half of the tumor was used for flow cytometry analysis, while the other half was used for histological analysis.

### Flow cytometry

Mice were euthanized at the endpoint, and tumors were collected. Flow cytometry was performed following the protocol mentioned in Knox et al. [12]. Briefly, isolated tumors were minced and subjected to tumor digestion using a buffer containing collagenase I, collagenase IV, hyaluronidase V, and DNAse I. Single cell suspension was washed thoroughly with PBS and incubated with Zombie Aqua™ Fixable Viability dye to discriminate dead cells. Cells were subjected to incubation with a panel of antibodies mentioned in Tables. S1 and S2 for myeloid cells and lymphoid cells. Data acquisition was performed on BD Celesta using FACS Diva software at GWU Flow cytometry core facility. Data analysis was performed using FlowJo software, and final plots and statistical analysis were performed with GraphPad Prism. Cell suspensions were labeled with CD45 and F4/80 antibodies for sorting tumor-infiltrated immune cells and collected live, double-positive cells for Isoplexis studies and CD45 + cells for 10xGenomics scRNA-seq analysis. Gating strategies for tumor macrophages and T-cells are depicted in Suppl. Fig. S9.

### Transcriptomic data analysis

For the analysis of M1 (*CD38*,* CD80*) and M2 (*ARG1*, *CD163*) related genes in melanoma, Kaplan-Meier overall survival was computed for the macrophage immune cell infiltrate in skin cutaneous melanoma (SKCM) dataset at The Cancer Genome Atlas (TCGA) database using web application Timer 2.0 (http://timer.cistrome.org/) [46]. The Outcome module allows users to explore the clinical relevance of tumor immune subsets (macrophages selected in this case) as a variable to determine the survival outcome based on the expression of M1 (CD38 and CD80) markers and M2 markers (ARG1 and CD163). Survival outcomes are computed using a multivariable Cox proportional hazard model. The Outcome module was used to compute the immune association between macrophage marker expression and tumor purity. The higher the infiltration of immune cells, the lower the tumor purity with an inverse correlation. The hazard ratio and p-value for the Cox model and the log-rank p-value for the Kaplan-Meier curve are calculated.

RNA-seq analysis of vehicle or HDAC6i, NextA treated BMDMs was performed as follows. FastQC (http://www.bioinformatics.bbsrc.ac.uk/projects/fastqc/) was used to evaluate the quality of the raw reads from M0, M1, and M2 samples in triplicates. The reverse and forward reads per sample were also assessed for paired-ended sequencing. The Mus musculus genome (GRCm39/mm39) was used as the reference for the transcriptomic quantification of the reads. Transcript quantification was performed with SALMON transcript quantification tool [47]. The reference genome served as the input index for the SALMON algorithm to report transcripts per mapped reads (TPM) per sample. The Empirical Analysis of Digital Gene Expression Data in R (EdgeR) package [46, 48, 49] in Bioconductor (version 3.40.1) was applied for differential expression analysis between M1 and M0, and between M2 and M0 in vehicle and NextA treatments. The weighted likelihood empirical Bayes method was used to control the p-values and the false discovery rate (FDR) [50]. The significance of gene expression was set at a threshold with p-value < 0.05, log2-fold-change < -1.5, or log2-fold-change > 1.5. A heatmap was used to visualize differentially expressed genes in various conditions. We used the Heatmapper tool [51], which applied the complete linkage method (DOI:10.1093/comjnl/20.4.364) combined with the Euclidian distance method (10.48550/arXiv.1502.07541) for gene clustering. To show the statistical significance of gene expression, volcano plots computing the log fold change and p-value using the web application VolcaNoseR were applied [52]. For pathway analysis, differentially downregulated genes after treatment with NextA were identified with a log2-fold-change < -1.5 and a p-value cutoff < 0.05 in the M1 and M2 macrophage groups. EnrichR functional analysis was performed on these genes using the BioCarta 2016 functional database terms. For gene set enrichment analysis (GSEA), the normalized transcript counts from each sample were uploaded to GSEA v4.3.3 and mapped to the MSigDB Hallmark Gene set collection (v2023.3). GSEA Enrichment scores were calculated by the degree of overrepresentation of a set of pathway-associated genes in the sample.

### Single-cell RNA-seq analysis

As mentioned above, single-cell suspension was prepared from tumors. Cell suspension was thoroughly washed with PBS and incubated with Zombie Aqua™ Fixable Viability dye, followed by incubation with anti-CD45.2 antibody conjugated to APC/Fire 750 fluorophore (Biolegend, Cat. No 109851) and sorted for live immune cells. The cell viability was verified to be above 95% before being subjected to 10X Genomics workflow. RNA-seq libraries were prepared following manufacturer’s instructions. The feature-barcode matrices generated by 10X Genomics CellRanger pipeline were analyzed through a standard workflow (Seurat v 4.0) in RStudio [53]. Cells with greater than 10% mitochondrial counts, fewer than 100 genes detected, or with a number of counts greater than the 93rd percentile of counts were removed. Mitochondrial genes were also removed. Cell type recognition was performed using the reference-based scRNA-seq annotation package, SingleR. The Immunological Genome Project (ImmGen) was used as the reference dataset [25]. The differential expression testing functionality of the Seurat package was used to examine differences in gene expression between two clusters of macrophages. The cluster of macrophages with higher expression of proinflammatory genes was classified as M1- macrophages, while the cluster with higher expression of anti-inflammatory genes was classified as M2-macrophages.

### Differential NicheNet analysis

Differential NicheNet analysis was performed using the Seurat object produced by the Seurat scRNA-seq workflow. Prediction of ligand-receptor pairs that are differentially expressed and active between different niches of interest (i.e., Control vs. M1 + NextA) was based on ligand-target gene regulatory potentials using publicly available data [54]. NicheNet predicted ligand-receptor interaction potential by searching protein-protein interactions between ligands and receptors. The interaction potential between ligands produced by the “sender” cell population (macrophages) and receptors expressed by the “receiver” cells (monocytes and T-cells) are summarized by the ligand-receptor network provided by NicheNet. Differential expression of ligands was calculated by comparing each sender cell type of the M1 + NextA niche to every sender cell type of the Control niche. Similarly, differentially expressed receptors between the receiver cell types were determined. The differential expression of ligands was summarized using the minimum log fold change (LFC) in the expression of the ligand when compared to the expression of that ligand in all sender cell types of the opposite niche. Ligand activities were predicted by determining a gene set of interest for each niche. For example, the gene set of interest for the M1 + NextA niche contained genes upregulated in the receiver cell population in the M1 + NextA group compared to the receiver cell population in the Control group. Target genes with an LFC greater than 0.15 were included in the gene sets of interest for each niche. Ligand-target links were then inferred based on the ligand-target matrix provided by NicheNet. Ligand activity was related to the upregulation of target genes in the receiver cells for each niche associated with each ligand. Average (scaled) expression and fraction of expression of ligands, receptors, and target genes are calculated across all cell types of interest. Next, ligand-receptor interactions were scored in a way that gives the highest score to the most highly expressed receptor of a certain ligand in a certain cell type. Ligand-receptor-target links are prioritized by calculating a weighted sum of the properties and weights described in the NicheNet. (https://github.com/saeyslab/nichenetr/blob/master/vignettes/differential_nichenet_pEMT.md). Figures were created for the top 30 prioritized ligands.

### Trajectory analysis

To understand the fate of tumor-infiltrated monocytes, we performed trajectory analysis based on the Monocle 3 tool in Partek Flow (Partek Inc). Briefly, the Seurat object was imported into Partek Flow with SingleR-defined cellular identities. This was followed by data normalization and scaling. Monocytes were manually defined as the root node and pseudotime analysis was performed to interrogate the fate of differentiation into macrophages in Control VS M1 + NextA tumors. Cell nodes are denoted as circles with numbers. The root node is a white circle, the branch node which represents a different cell fate, is denoted as a black circle, and finally leaf represented as a grey circle is the differentiated cells state. MSigDB Hallmark 2020 pathway and GO biological process analysis of top 100 significant genes based on p values was performed on Enrichr (https://maayanlab.cloud/Enrichr/) [55].

### Single-cell secretome analysis

Isoplexis platform was used to perform sc-secretome analysis on mouse and human innate immune IsoCode chips. As described earlier, murine and human macrophages were derived from bone marrow and monocytes. The macrophages were either treated with vehicle or NextA prior to polarization to M1 and M2 phenotypes. Macrophages were stained in plates with CellStain 405 at a dilution of 1:500 for 20 min, followed by washing with a complete medium. Stained cells were gently scrapped to dislodge from cell culture plates, and the cell number was adjusted to 750,000 cells/mL. TAMs were obtained from tumors of vehicle and adoptive cell therapy treated mice by flow sorting for CD45+ (APC/Fire™ 750 anti-mouse CD45.2) and F4/80 + cells (Alexa Fluor 647 anti-mouse F4/80). Macrophages were loaded into respective IsoCode chips and analyzed on IsoSpark. Run data was analyzed with IsoSpeak software to obtain a sc-secretome of M0, M1, and M2 macrophages.

### Immunoblot analysis

Cells were lysed in RIPA buffer (Thermo Scientific, Cat# 89900) supplemented with protease and phosphatase inhibitor (Thermo Scientific, Cat# 78440). Equal concentrations were loaded on 4–20% gradient SDS-PAGE gels (Bio-Rad, Cat# 456–1093) after protein estimation by BCA assay. Proteins were transferred onto low fluorescence PVDF membranes (Bio-Rad, Cat# 1704274) using a Trans-Blot Turbo transfer system (BioRad). The membranes were blocked for one hour with Odyssey blocking buffer (LI-COR, Cat# 927-40000), followed by incubation with primary antibodies (1/1000 dilution) at 4 °C. The membranes were washed in PBST buffer (3x), followed by incubation with near-infrared fluorophore-conjugated secondary antibodies (1/10,000 dilution) for 1 h at room temperature. The membranes were scanned on an Azure Biosystems c600 imager at near-infrared wavelengths. The images were analyzed and processed with Image Studio Lite software. The primary antibodies used are against phospho-STAT3 Y705 (Cell Signaling, 9145), α-tubulin (Cell Signaling, 3873), acetyl-α-tubulin (Cell Signaling, 3971), histone H3 (Cell Signaling, 3638), acetyl-histone H3 (Cell Signaling, 9649), HDAC6 (Assay Biotech, C0226), arginase 1 (Cell Signaling, 93668), and iNOS (Invitrogen, PA3-030 A).

### Quantitative real-time PCR

Total RNA was isolated following the manufacturer’s instructions using Trizol (ThermoFisher Scientific, Cat#15596026). For cDNA synthesis, 1ug of total mRNA was subjected to reverse transcription using an iScript cDNA synthesis kit (Bio-Rad, Cat#1708891). Real-time quantitative PCR was performed on Bio-Rad CFx Differential gene expression analysis was performed by 2^–∆∆Ct^ method normalized to reference gene which is *GAPDH* or *ACTB* unless otherwise specified. The list of primers used in this study are listed in Tables. S3 and S4.

### Assessment of viability

Bone marrow-derived macrophages (BMDMs) were isolated as previously described. BMDMs were left unpolarized and treated with different concentrations of the HDAC6i NextA or the pan-HDACi Panobinostat as a positive control. After 24 h of treatment, macrophages were gently scraped and stained with viability dye Live/dead™ Fixable Aqua Dead Cell Stain Kit (Invitrogen, L34966). Data was acquired on BD Celesta using FACS Diva software and analyzed on FlowJo software.

### shRNA transfection

To decrease the expression of HDAC6 in BMA3.1A7 cells, murine shRNA for HDAC6 (SHCLND-NM_010413, Sigma-Aldrich) was packaged into lentiviral particles and virally transfected into macrophages along with a non-target control vector. The knockdown clones were cultured under antibiotic selection, and HDAC6 knockdown was verified by western blot.

### Histology

Immunohistochemistry (IHC) and Hematoxylin and Eosin staining of formalin-fixed paraffin-embedded tissue sections and immunofluorescence (IF) staining of paraformaldehyde-fixed murine cells were conducted using standard protocols. For antigen-retrieval, slides were boiled in citrate-based antigen unmasking buffer (Vector Labs) or Tris-based pH9 antigen unmasking buffer (Vector Labs) for 10 min in the microwave. Slides were incubated with primary antibodies at 4 °C overnight. For multi-color IHC, sequential staining was conducted, with Avidin-Biotin blocking (Life Technologies) between each stain. Primary antibodies and dilutions were rabbit anti-GFP (Cell Signaling 2956; 1/200), chicken anti-GFP (Abcam ab13970; 1/1000), rat anti-CD206 (Biolegend 141701; 1/200), and rabbit anti-CD38 (Abcam ab216343; 1/1000). To detect human antigens, the primary antibodies and dilutions were mouse anti-CD206 (Biolegend 321101; 1/200) and rabbit anti-CD38 (Abcam ab108403; 1/1000). ABC Elite (Vector Labs), Vector SG (Vector Labs), and NovaRED Substrate (Vector Labs) kits were used for signal detection of IHC staining. Alexa Fluor secondary antibodies were used for IF staining. Nuclei were counterstained with Fast Red (StatLab), Hematoxylin (Fisher), or DAPI (Invitrogen). Staining was imaged on Leica DMi8 and Zeiss 710 confocal microscopes for IHC and IF experiments, respectively.

### Macrophage and T-cell co-culture assay

BMDMs were isolated and pretreated with HDAC6i NextA as mentioned above. On day 6 after BMDM isolation and phenotype polarization, T-cells from the mouse spleen were isolated with EasySep™ Mouse T Cell Isolation Kit (Stemcell Technologies, Cat # 19851) following the manufacturer’s instructions. Isolated T-cells were labeled with CellTrace™ Violet Cell Proliferation Kit (ThermoFisher, Cat # C34557) and subsequently activated with Dynabeads™ Mouse T-Activator CD3/CD28 for T-Cell Expansion and Activation (ThermoFisher, Cat # 11452D) in RPMI complete media supplemented with 100U/mL of mouse recombinant Il-2 cytokine (ThermoFisher, Cat # 212 − 12) as per the manufacturer’s recommendations. M0, M1, and M2 BMDMs treated with or without NextA were gently harvested using a cell scrapper. Macrophages and activated T-cells were incubated at a 1:2 ratio for 72 h to determine T-cell proliferation. Media was collected from the co-culture assay and analyzed for cytokine expression profile using Mouse adaptive immune Codeplex secretome chips (Isoplexis) on the IsoSpark system. T-cells co-cultured with macrophages were stained with anti-mouse antibodies CD4-BV650, CD8-APC-Fire 750, and propidium iodide live dead stain for flow cytometry analysis on BD LSRFortessa. Data analysis was performed on FlowJo and graphical representation on GraphPad Prism software.

### Statistical analysis

All statistical analyses were performed using GraphPad Prism software (Version 10.1.1). The continuous variables were represented as the median or mean with standard deviation where appropriate. Wherever appropriate, non-parametric unpaired t-test or one-way ANOVA was performed for continuous variables using the multiple comparisons function. Survival analysis was performed using the Kaplan-Meier method, and survival curves were compared for significance using the log-rank test. Unless otherwise specified, a p-value less than 0.05 was considered statistically significant for all statistical analyses.

## Electronic supplementary material

Below is the link to the electronic supplementary material.

Supplementary Material 1

## Data Availability

The datasets used and/or analyzed during the current study are available from the corresponding author upon reasonable request.
