# Supplementary material for: Cell therapy using ex vivo reprogrammed macrophages enhances antitumor immune responses in melanoma
Source: J Exp Clin Cancer Res. 2024 Sep 14;43:263. doi: 10.1186/s13046-024-03182-w (PMC11401321; doi:10.1186/s13046-024-03182-w)
Supplement: Supplementary file 1 — Supplementary Material 1 [file 13046_2024_3182_MOESM1_ESM.docx]

**
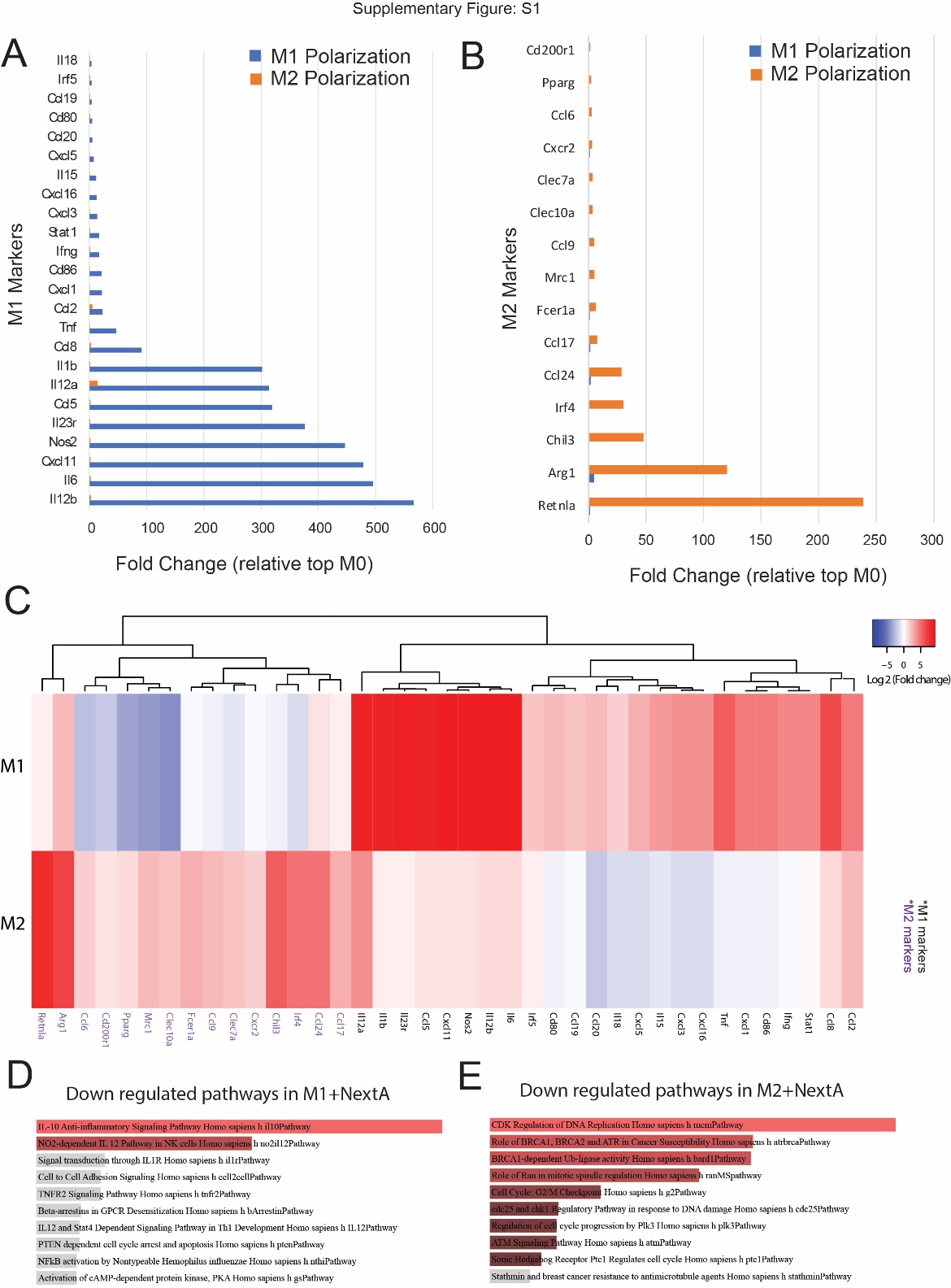
**

**Supplementary Figure. S1. Top genes down- or upregulated in macrophages treated with HDAC6i.** (A-B) Expression levels of M1 specific markers and M2 specific markers in macrophages are represented as fold changes relative to M0. (C) Heatmap representing unsupervised clustering of M1 and M2 markers as log 2-fold change relative to M0 where red indicates higher expression and blue indicates lower expression. (D) Pathways down regulated in M1 macrophages treated with NextA. (E) Pathways down regulated in M2 macrophages treated with NextA. Pathway analysis was performed with Enrichr.

**
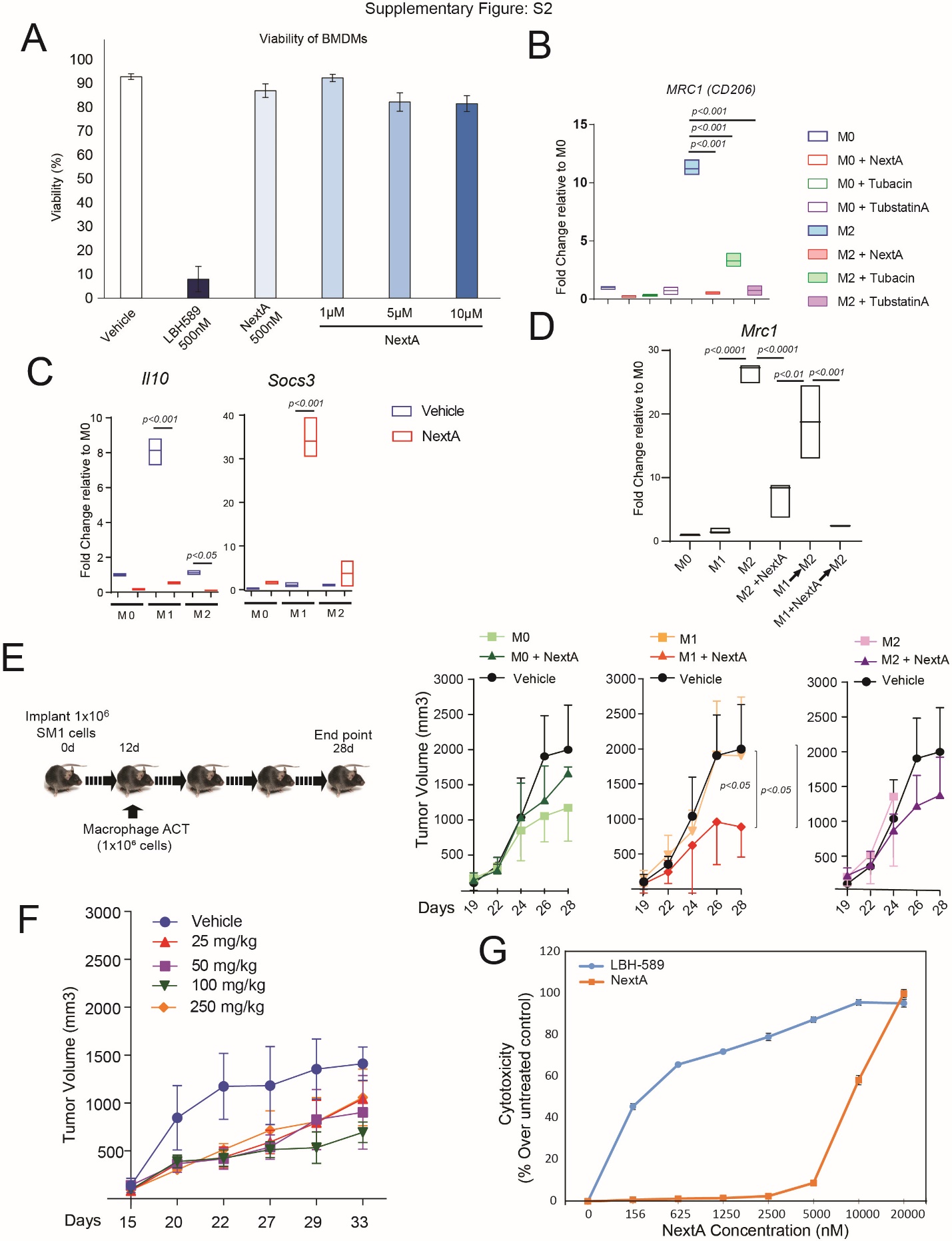
**

**Supplementary Figure. S2. Dose titration analysis and preliminary ACT therapy.** (A) BMDMs were treated with an increasing concentration of NextA to validate the cytotoxicity using pan-HDAC inhibitor LBH589 as a positive control. (B) Expression of M2 marker *MRC1* (CD206) in Thp1 derived macrophages treated with various HDAC6 inhibitors NextA, tubacin, and tubastatin A. Gene expression analysis by qRT-PCR and represented as fold change relative to M0 (C) BMDMs polarized to M1 and M2 compared to naïve macrophages M0 by qRT-PCR. Analysis of *Il10* and *Socs3* genes. (D) M1 to M2 repolarization assay with RAW 264.7 macrophages. Expression of *Mrc1* (*Cd206*) determined by qRT-PCR. (E) SM1 intratumor adoptive transfer of murine BMDMs. Tumor growth charts of SM1 melanoma tumors after syngeneic adoptive cell transfer of 1x10^6^ M0, M1, and M2 macrophages treated with vehicle or NextA. (n=10 mice/group) (F) Intra-tumor dose titration of NextA in SM1 murine melanoma model. Intra-tumor injection of NextA at indicated concentrations indicated that 100µg was an effective dose. (n=5 mice/group) (G) Cytotoxicity assay of SM1 murine melanoma performed with NextA and pan-HDAC inhibitor LBH-589 (Panobinostat) at indicated concentrations ranging from 0 to 20µM.

**
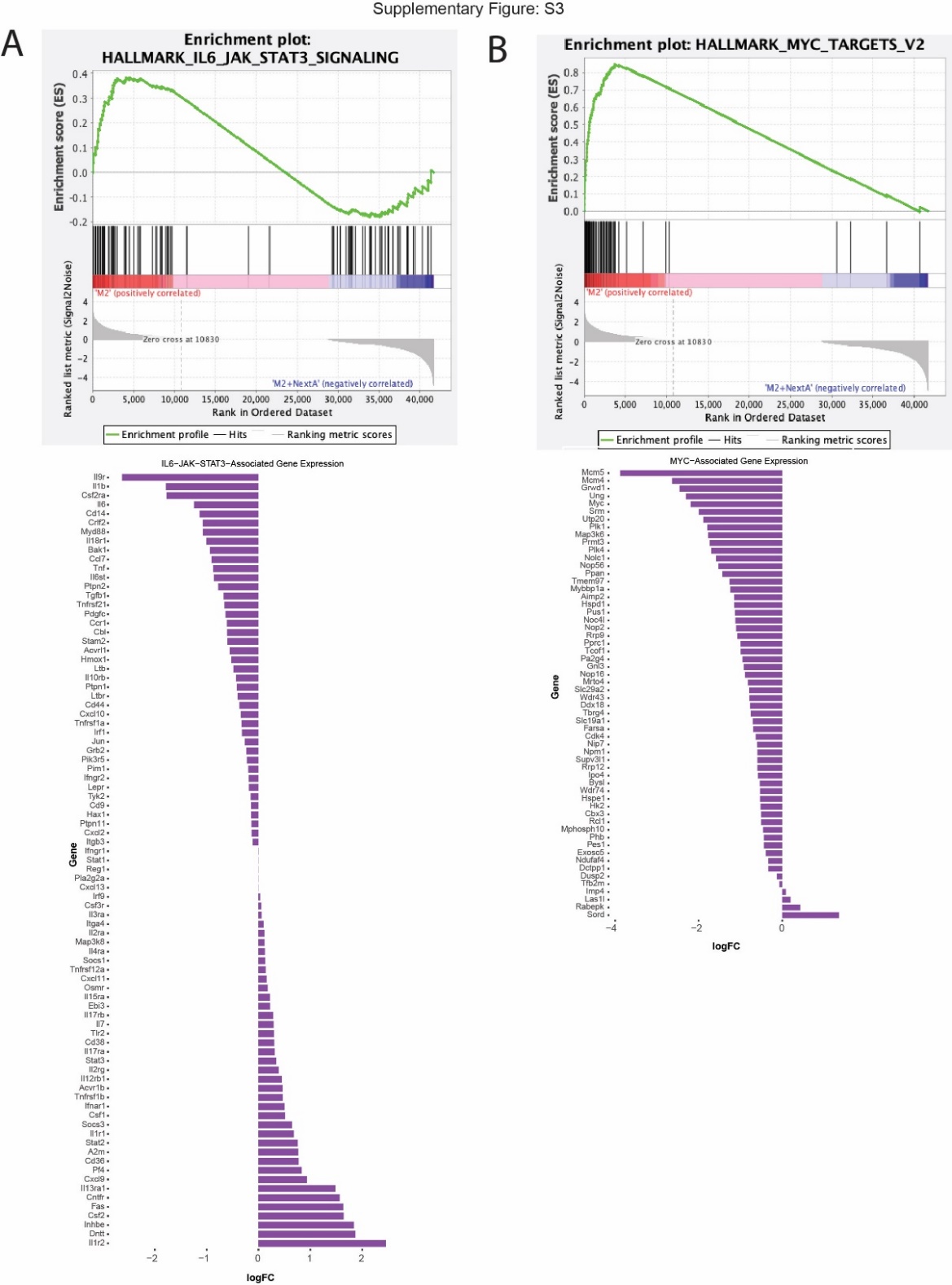
**

**Supplementary Figure. S3. Suppression of M2 polarizing pathways with HDAC6 inhibition.** Gene set enrichment analysis of M2 and M2+NextA transcriptomes. (A) Enrichment plot indicating suppression of IL6-JAK-STAT3 axis in M2+NextA macrophages. Fold changes of genes in the pathway are represented by a blot plot. (B) Enrichment plot indicating suppression of Myc targets in M2+NextA macrophages. Fold changes of genes in the pathway are represented by a blot plot.

**
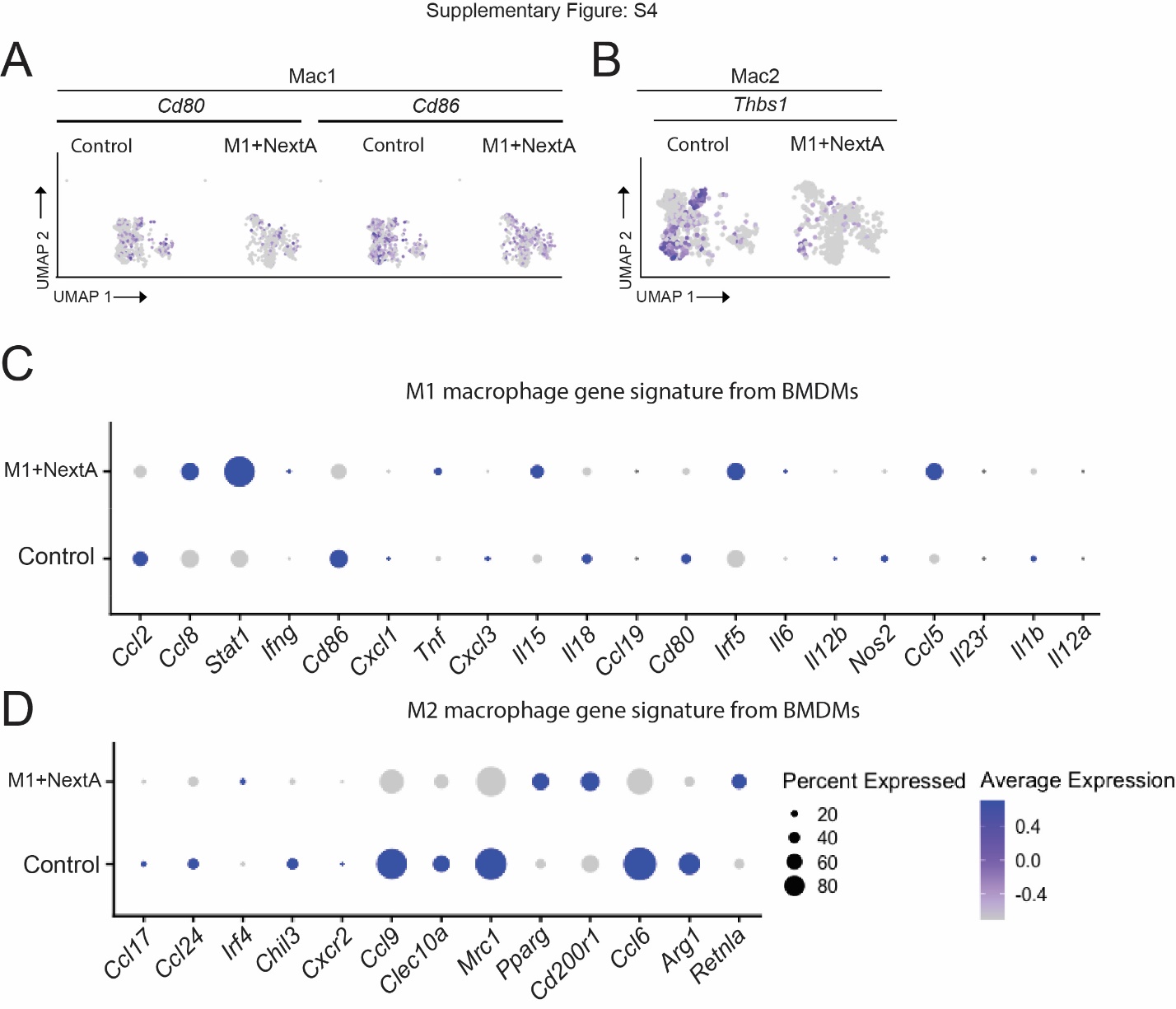
Supplementary Figure. S4. Expression of M1 and M2 gene signature in Control and ACT tumors.** (A) Feature plots representing expression of inflammatory genes *Cd80* and *Cd86* associated with M1 phenotype in Mac1 macrophage subcluster in Control and M1+NextA tumors. (B) Feature plots representing expression of tumor promoting genes *Thbs1* associated with M2 phenotype in Mac2 macrophage subcluster in Control and M1+NextA tumors. (C) Upregulation of M1 signature in M1+NextA tumor and (D) M2 gene signature in Control tumor.

**
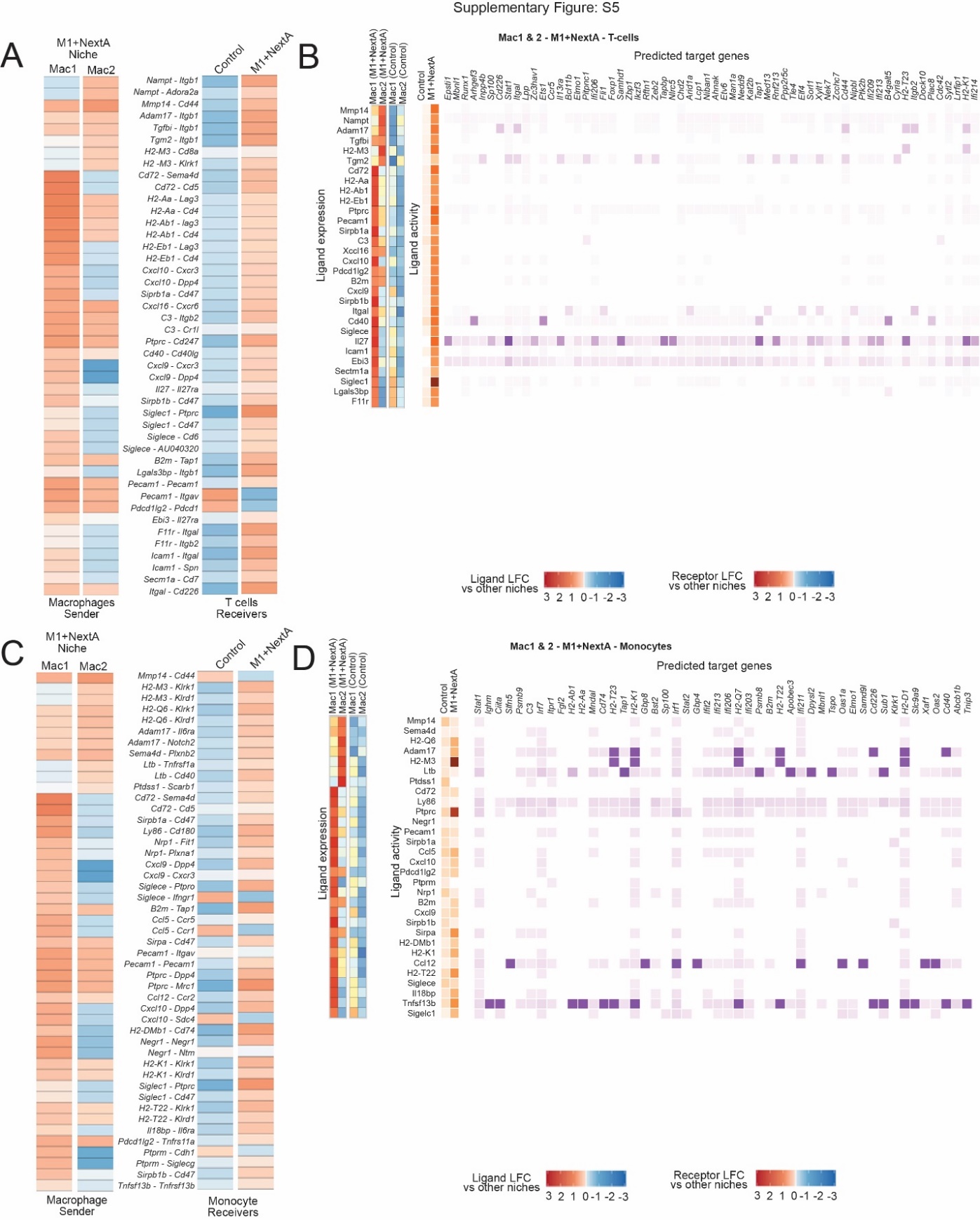
**

**Supplementary Figure. S5. Cell-cell interactions between tumor macrophages and T-cells and monocytes indicate activation of inflammatory response in M1+NextA tumors.** In differential NicheNet analysis, macrophages from Mac1 and Mac2 subclusters were designated as senders of ligands to the receptors expressed on receiver T-cells, and monocytes. (A) Heatmap representing top 30 ligand-receptor interaction pairs differentially expressed between macrophages in Mac1 and Mac2 senders in the M1+NextA tumor and T-cell receivers. (B) Heatmap of ligand activity and projected target genes activated in receiver T-cells in M1+NextA tumor. The top 30 ligands were scored based on the correlation coefficients and regulatory potential scores of their target genes in the receiver cells. (C) Heatmap representing top 30 ligand-receptor interaction pairs differentially expressed between macrophages in Mac1 and Mac2 senders in the M1+NextA tumor and monocyte receivers. (D) Heatmap of ligand activity and projected target genes activated in receiver monocytes in the M1+NextA tumor.

**
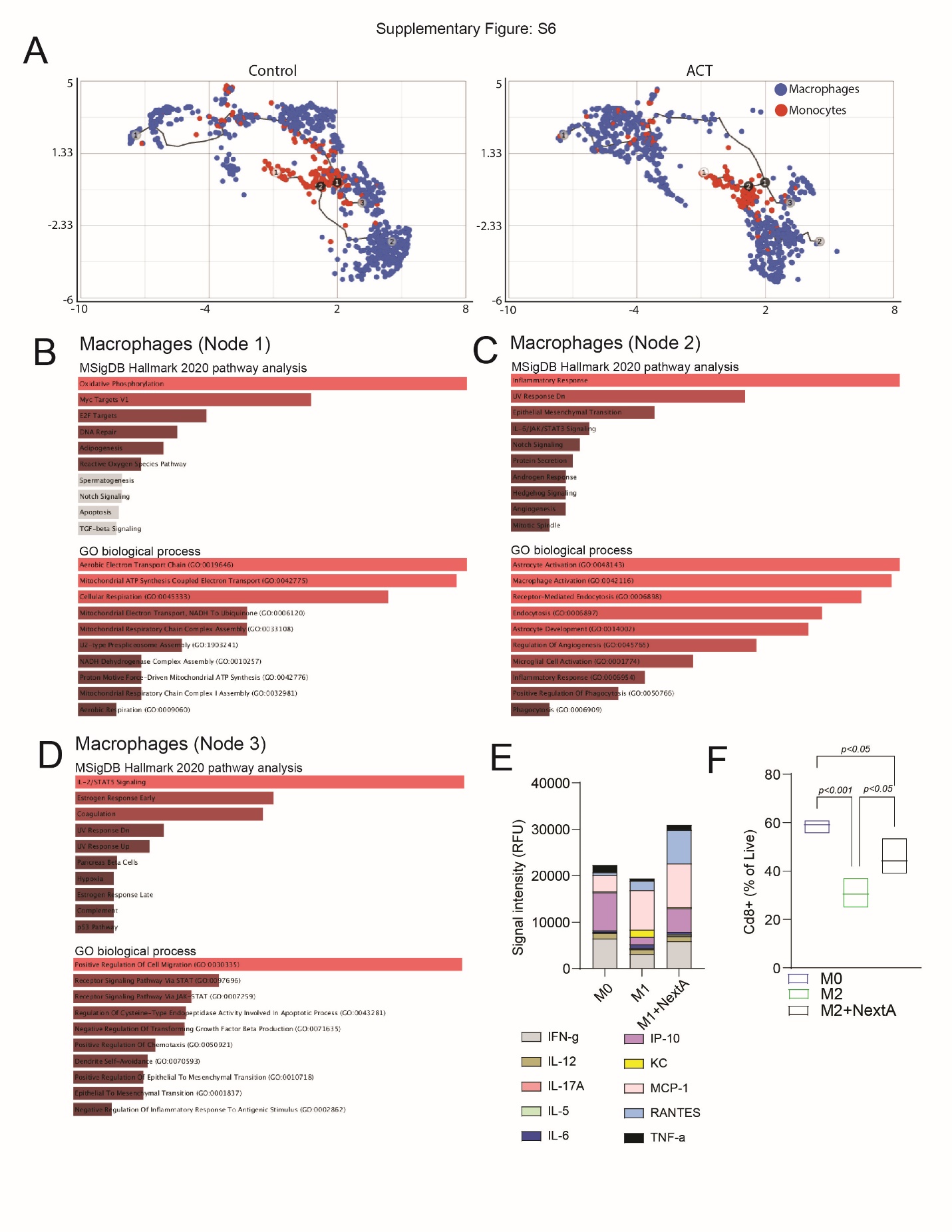
Supplementary Figure. S6. Trajectory analysis indicated the transitioning of tumor monocytes into inflammatory macrophages in M1+NextA tumors.** Monocle 3-based pseudotime trajectory was used to track the fate of monocytes in the TME. Monocytes were selected as the root node that branched off into a transitioning cluster of monocytes (indicated with black circles) and three predominant clusters of differentiated macrophages (grey circles with numbers inside). The macrophage clusters will be referred to as Nodes 1, 2, and 3. (A) Pseudotime analysis of tumor infiltrated monocytes (indicated as red dots) transitioning into tumor macrophages (blue dots). Monocytes are designated as the root node (white circle) with branch-off nodes (black circles) giving rise to differentiated macrophages (grey circles). The black line indicated the trace of trajectory on a pseudotime scale. (B) Pathway analysis using the MSigDB Hallmark 2020 module and GO biological process module of Enrichr with top 100 significant genes based on p values in macrophage Node 1. Similar analyses were performed on macrophage Nodes 2 (C) and 3 (D). (E) Adaptive immune cytokine and chemokine secretome profile of T-cells co-cultured with M0, M1, and M1+NextA macrophages analyzed on the Isoplexis platform. (F) Flow cytometry analysis of CD8 T-cell proliferation co-cultured with M0, M1, and M1+NextA macrophages. T-cells were stained with CellTrace Violet dye.

**
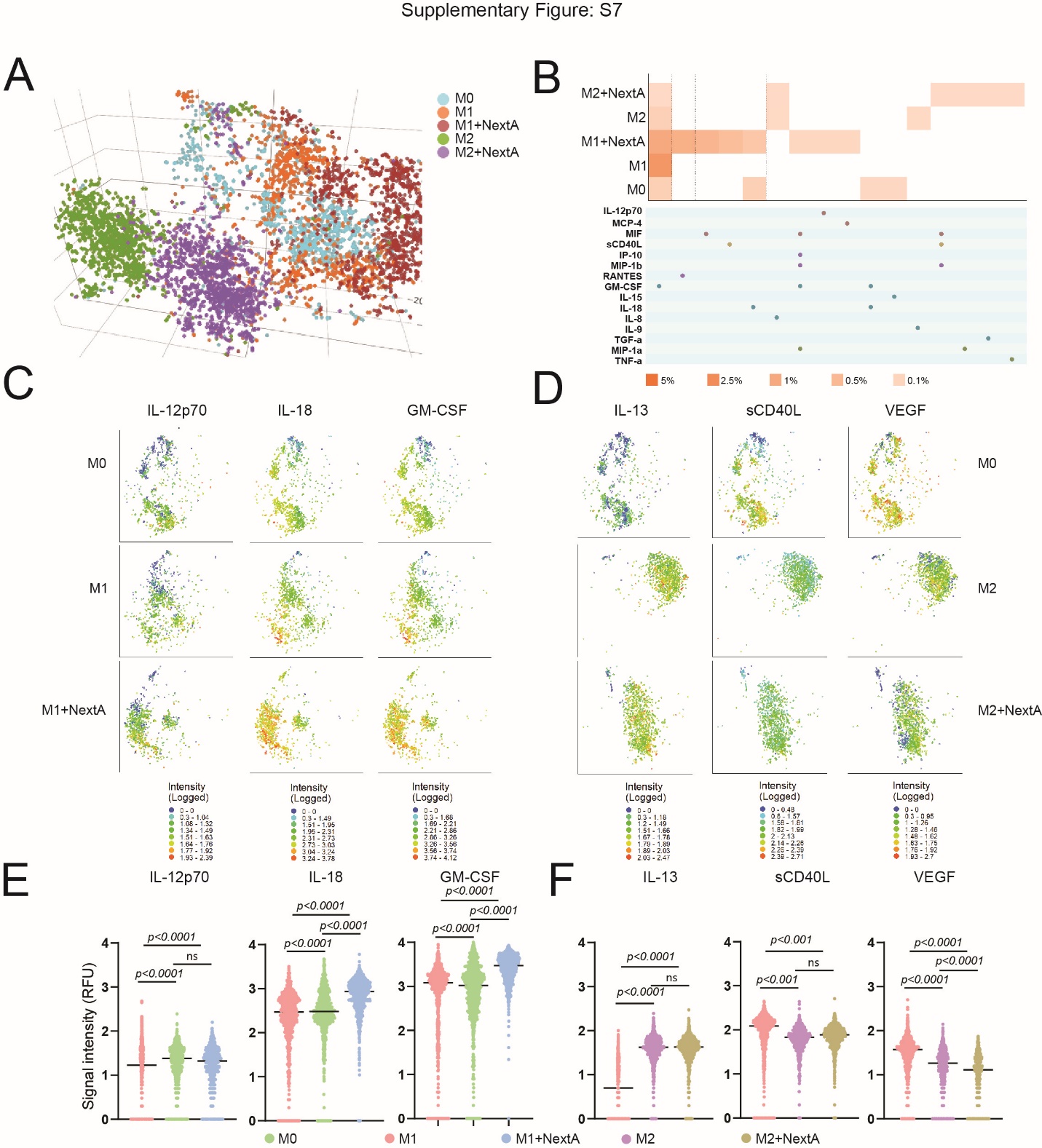
**

**Supplementary Figure. S7. Human macrophage single cell secretome analysis.** (A) Singel-cell secretome analysis of human macrophages. 3D-Tsne plot of human monocyte-derived macrophages at a single cell resolution separated the cells into defined clusters based on their secretome profile. About 1226 M0, 1129 M1, 1177 M1+NextA, 1342 M2, and 1407 M2+NextA treated human macrophages were analyzed on human innate immune IsoCode chips. (B) Polyfunctionality heatmap showing M1+NextA macrophages secreting more than one cytokine/chemokine compared to other phenotypes. 2-D tsne plots where each cell is represented as a colored dot. As shown in the intensity scale, blue indicates low expression, and red indicates high expression. Scatter plots of respective cytokines are represented as signal intensity. (C) 2D-Tse plots of proinflammatory cytokines IL-12p70, GM-CSF and, IL-18 in M1+NextA compared to M1 and M0 and shown as scatter plots in (E). (D), 2D-Tsne plots of immunoregulatory IL-13, sCD40L, and VEGF in M2 macrophages compared to M0 and M2 macrophages and shown as scatter plots in (F).

**
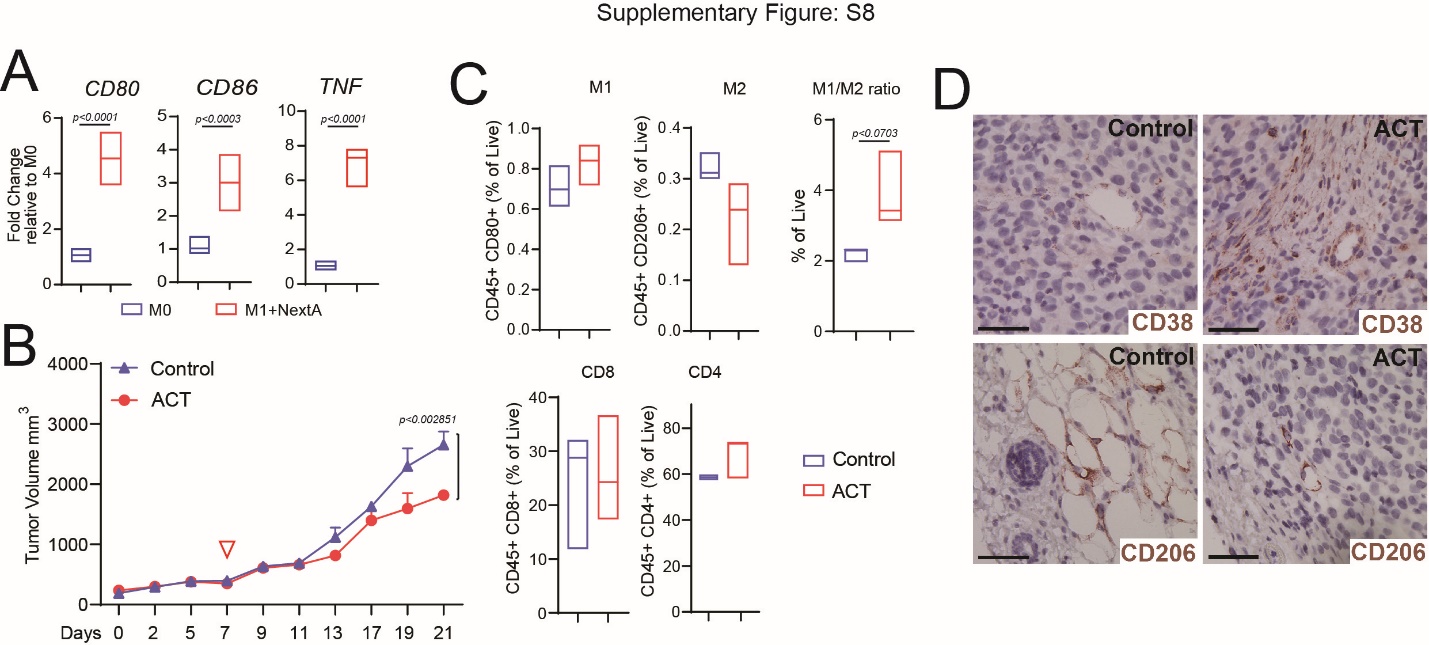
**

**Supplementary Figure. S8. Macrophage adoptive cell therapy recapitulated antitumor effect in humanized mouse model (**A) Validation of M1 genes *CD80* and *CD86* in human monocyte-derived macrophages prior to ACT with M1+NextA macrophages (n=5 mice/group). (B) NSG-SGM3 humanized model treated with PBS (Control) partial HLA-matched NextA treated M1 macrophages (2x10^6^) intratumor ACT. Tumor growth kinetics of melanoma patient-derived xenograft (PDX) tumors in NSG-SGM3 mice in control and treatment groups (n = 3). (C) Immunophenotyping of tumor-infiltrated immune cells by flow cytometry. CD80+ M1 macrophages represented as a percentage of CD45+ immune cells and CD206+ M2 macrophages represented as a percentage of CD45+ immune cells. (D) Immunohistochemistry analysis of PDX tumors post ACT therapy for M1 macrophages (CD38) and M2 macrophages (CD206).

**
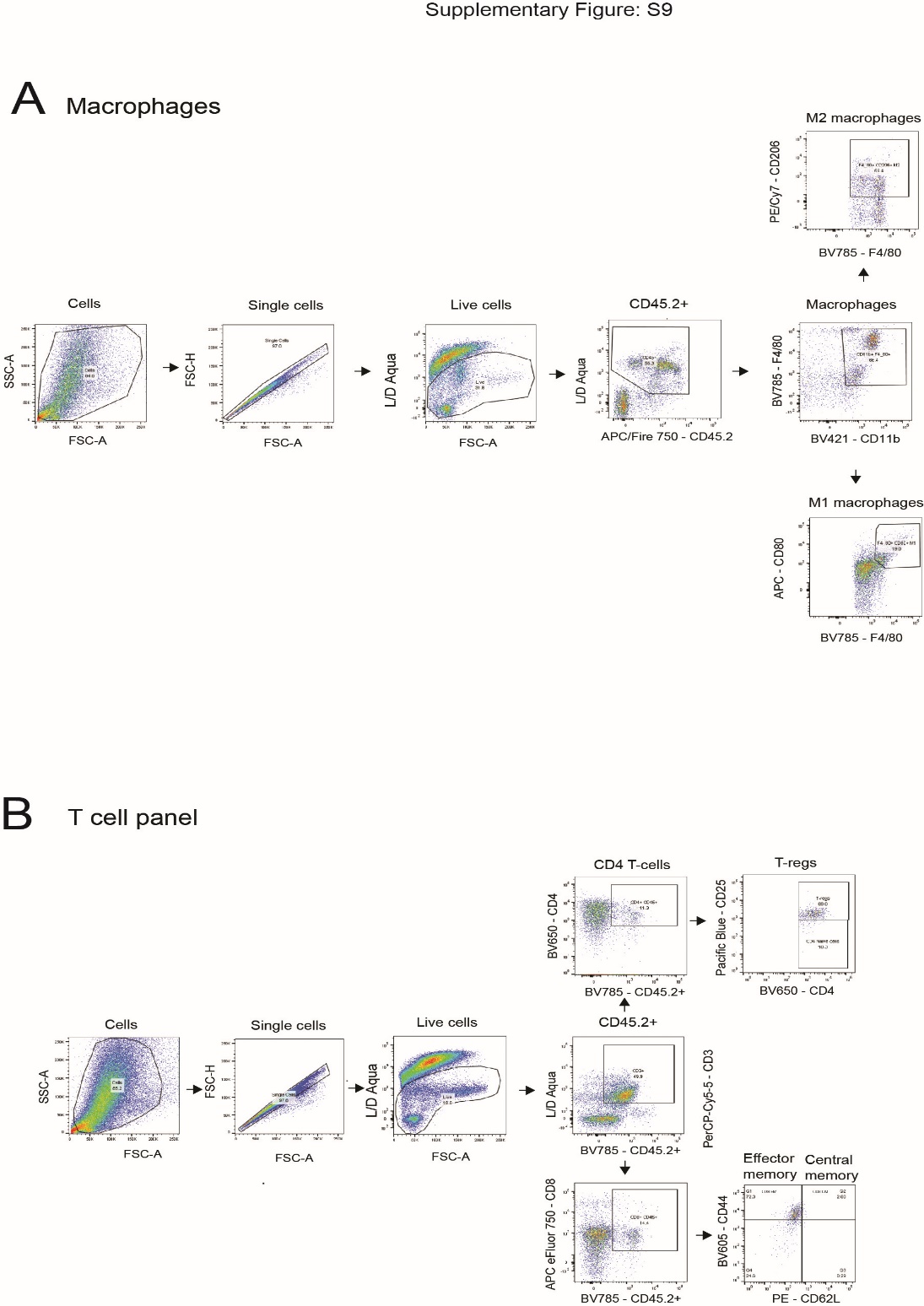
**

**Supplementary Figure. S9. Gating strategy for flow cytometry analysis. (**A) Gating strategy for flow cytometry analysis of macrophages and (B) T-cells.
